# A flexible dose-response modeling framework based on continuous toxicity outcomes in phase I cancer clinical trials

**DOI:** 10.1186/s13063-023-07793-0

**Published:** 2023-11-21

**Authors:** Se Yoon Lee

**Affiliations:** Department of Statistics, Texas A &M University, 3143 TAMU, College Station, 77843 TX USA

**Keywords:** Dose-response model, Continuous toxicity outcomes, Bayesian adaptive designs, Phase I cancer clinical trials

## Abstract

**Background:**

The past few decades have seen remarkable developments in dose-finding designs for phase I cancer clinical trials. While many of these designs rely on a binary toxicity response, there is an increasing focus on leveraging continuous toxicity responses. A continuous toxicity response pertains to a quantitative measure represented by real numbers. A higher value corresponds not only to an elevated likelihood of side effects for patients but also to an increased probability of treatment efficacy. This relationship between toxicity and dose is often nonlinear, necessitating flexibility in the quest to find an optimal dose.

**Methods:**

A flexible, fully Bayesian dose-finding design is proposed to capitalize on continuous toxicity information, operating under the assumption that the true shape of the dose-toxicity curve is nonlinear.

**Results:**

We conduct simulations of clinical trials across varying scenarios of non-linearity to evaluate the operational characteristics of the proposed design. Additionally, we apply the proposed design to a real-world problem to determine an optimal dose for a molecularly targeted agent.

**Conclusions:**

Phase I cancer clinical trials, designed within a fully Bayesian framework with the utilization of continuous toxicity outcomes, offer an alternative approach to finding an optimal dose, providing unique benefits compared to trials designed based on binary toxicity outcomes.

## Background

Phase I cancer clinical trials are a critical first step in the study of novel cancer therapeutic approaches. One of the primary goals of the phase I studies is to determine the dose of a new drug or therapeutic agent for use in subsequent phase II trials [1–4]. In these trials, one of the fundamental assumptions is that toxicity is a precondition for anti-tumor activity to eliminate fast-growing cancer cells [5]. This means that patients must endure some degree of treatment-related toxicity to have a reasonable chance of a favorable response. More precisely, the purpose of the cancer phase I clinical trial is to estimate the maximum tolerated dose (MTD) of a new drug associated with an acceptable level of dose-limiting toxicity (DLT) [6].

The use of model-based adaptive clinical trial designs in phase I clinical trials has received much attention because it allows adaptations of trials and statistical designs of ongoing clinical trials [7–9]. Most designs developed in the literature consider binary toxicity outcomes, but recently, there has been increasing recognition of the need to identify the MTD by incorporating continuous toxicity information. One motivation for this is that some binary toxicity outcomes are obtained by dichotomizing continuous data, inevitably incurring losses of information and statistical power in clinical trial designs [10–12]. This convention imposes an additional burden of finding an optimal cutpoint for the continuous data and may require more subjects than if the endpoint variable were utilized in its original continuous form [13].

The other motivation is that various continuous measures of toxicity arise during phase I studies, which can be beneficial for examining dose-response relationships more precisely. For example, if one aims to consider all grades of toxicities from multiple adverse events when allocating doses, it may be necessary to derive a continuous toxicity score, such as the normalized equivalent toxicity score [14, 15], or use a weighted average form as discussed by [16], and utilize such scores as the continuous toxicity response.

Additionally, in pursuit of efficiency and speed in drug development, trialists are increasingly relying on the use of real-world data to assess the potential risks and benefits of new drugs, and much of this data may be originally measured on a continuous scale [17]. One example is the drug concentration in plasma from patients [18, 19]. It is widely known that overly high anti-cancer drug concentrations may be a risk factor for many side effects, such as cytokine release syndrome [20–22], and therefore, the drug exposure may represent a toxic reaction, depending on what is known about the mechanism of action of the drug. Another example is a logarithmic transformation of the number of white blood cells, which serves as a continuous toxicity response [23]. Furthermore, any quantity measured in real numbers, as a measurable indicator of the severity of some toxicity status related to new drug exposure, may be used as a surrogate endpoint, provided that a higher value of the quantity leads to a higher probability of side effects for patients as a price for a higher chance of treatment effect [24, 25].

Over the past few decades, there has been a remarkable development in adaptive dose-finding designs for phase I studies using binary toxicity outcomes, such as the continual reassessment method [26], the escalation with overdose control (EWOC) [3], and the Bayesian logistic regression model [27], along with their several extensions [28–32]. Refer to [4] for a survey of these methods. A theoretical framework using the binary toxicity response can be found in [33].

However, the utilization of continuous toxicity outcomes for optimal dose finding has garnered relatively little attention compared to its counterpart based on binary toxicity outcomes. Since the earliest research work by Eichhorn in the 1970s [34], only a handful of research studies have been published [35], and there is no established theoretical framework available to develop a model-based design in the literature. Recently, Chen et al. [15] proposed a variant version of EWOC, named EWOC-NETS, based on a pseudo-Bernoulli likelihood [36], where the binary toxicity response is replaced with a continuous fractional response derived from a toxicity score system. One drawback of EWOC-NETS is that the dose-finding does not follow the fully Bayesian paradigm, thus failing to describe the uncertainty of the MTD in a fully Bayesian manner. More recently, Lee et al. [16] introduced a fully Bayesian design based on constrained linear regression, called the two-parameter linear dose-finder. In this design, the authors aimed to leverage all grade information of toxicities according to the Common Toxicity Criteria for Adverse Events (CTCAE) [37] within a fully Bayesian framework. Although the design provides a fully Bayesian dose-finding algorithm, it falls short in describing the non-linear shape of the dose-toxicity curve, and its application is confined to analyzing grade information based on CTCAE.

In this paper, our objective is to bridge the existing gap by introducing a novel, fully Bayesian dose-finding algorithm that provides flexibility in describing the dose-toxicity curve and wider applicability, based on continuous outcomes. The incorporation of a continuous response within our framework accommodates various scenarios, such as using a continuous toxicity score [16], measuring drug concentration in plasma from patients [18], a biomarker response from a molecularly targeted agent [38], and so on.

## Methods

This section presents a general modeling framework and performance evaluation metrics for dose-finding designs that utilize continuous toxicity responses, which are widely applicable in the context of phase I cancer trials. Following this, we introduce a new fully Bayesian design aiming to estimate the MTD or an optimal dose under a specific setting where the dose-response curve is non-linear.

### Continuous toxicity response

We start by characterizing some basic concepts that define a continuous toxicity response in the dose-response modeling framework. To that end, we first need to “clinically” understand two fundamental concepts: MTD and DLT, which are central to dose-finding problems in phase I trials. The mathematical definitions of MTD and DLT will be discussed in the next subsection.

The aims of typical phase I oncology trials are to determine MTD, assess the safety and tolerability, and investigate the pharmacokinetics and pharmacodynamics of a new drug. The United States National Cancer Institute defines MTD as the highest dose of a drug that does not cause unacceptable side effects (Visit the website www.cancer.gov/- for the definition). The determination of the MTD is based on the occurrence of DLT. DLT refers to drug-induced toxicity associated with side effects of a drug that are serious enough to prevent an increase in dose. A clinical trial protocol must specify the criteria for DLT, often defined as any severe or life-threatening adverse event [4].

Throughout the paper, *x* represents a dose of a new anti-cancer drug. We denote *Y*(*x*) to represent the continuous toxicity response of a patient against dose *x*. Technically, the dose *x* is an input with a positive real number (that is, $x\in (0,\infty )$), and the response *Y*(*x*) is an output assuming a real number. Allowing the response *Y*(*x*) to attain a negative real number is important for the generality of the dose-finding problems because, in many pharmaceutical applications, *Y*(*x*) may represent a change from baseline or a log-transformation of some continuous measurement. Eventually, for each patient, the outcome of the trial is represented by the ordered pair $(x,Y(x)) \in (0,\infty ) \times \mathbb {R}$.

We denote an open interval $(x_{\text {min}}, x_{\text {max}}) \subset (0,\infty )$ to represent an admissible dose range that clinicians want to explore during the trial. The dose range is determined by clinicians and remains fixed during the trials. Although clinicians expect that MTD would belong to the interval $(x_{\text {min}}, x_{\text {max}})$ (i.e., $\text {MTD} \in (x_{\text {min}}, x_{\text {max}})$), MTD is an unknown quantity, and we do not know whether it will indeed fall within this interval (i.e., it could happen that $\text {MTD} \notin (x_{\text {min}}, x_{\text {max}})$). Statistically, MTD is the parameter of main interest in phase I studies. Given no toxicity information from any patient, all we can do is widen the range of the interval $(x_{\text {min}}, x_{\text {max}})$ to increase the probability of MTD falling within the dose range. This comes at the price of decreasing the power of a model, as commonly encountered in many statistical problems.

Phase I trial designs for cytotoxic agents are based on the assumptions that (a) the clinical benefit of the agent increases with increasing dose, (b) the toxicity of the agent increases with increasing dose, and (c) there is a dose with acceptable toxicity that offers clinical benefit [39]. With these assumptions in mind, we posit five assumptions that define the continuous toxicity response *Y*(*x*). Later on, we will see that these assumptions are essential in bringing the clinical concepts of MTD and DLT to a statistical model-based design:

- A1. A side effect caused by the toxicity response *Y*(*x*) is negligible for doses $x\in (0,x_{\text {min}})$.
- A2. A side effect caused by the toxicity response *Y*(*x*) is mild at the dose $x_{\text {min}}$.
- A3. A side effect caused by the toxicity response *Y*(*x*) becomes more and more serious as the dose increases over the interval $(x_{\text {min}}, x_{\text {max}})$.
- A4. A side effect caused by the toxicity response *Y*(*x*) at the dose $x_{\text {max}}$ is life-threatening or close to death.
- A5. A side effect caused by the toxicity response *Y*(*x*) is too fatal for doses $x \in (x_{\text {max}},0)$.

The assumptions above are common features describing a toxicity response used in many phase I clinical trials [5, 27, 40]. Many binary toxicity outcomes may be obtained by dichotomizing a continuous toxicity outcome ($\text {DLT if }$ $Y(x) > \eta ;$ ,  $\text {non-DLT otherwise}$, provided a constant $\eta$) [35]. Regarding the statements in the assumptions, the terms “mild,” “life-threatening,” and “death,” indicating the severity of toxicity, can refer to the United States National Cancer Institute’s Common Toxicity Criteria (CTCAE) [37]. However, these terms can be generalized depending on the context of the therapeutic area or the safety guidelines from regulatory agencies for drug approval, as long as the monotonic relationship between the response *Y*(*x*) and dose *x* holds.

The following are some important implications based on the assumptions. The first assumption (A1) means that patients are expected to have no treatment effect when they are assigned a dose $x\in (0,x_{\text {min}})$. Thus, doses $x\in (0,x_{\text {min}})$ are not going to be explored in phase I clinical trials. The second assumption (A2) implies that the dose $x_{\text {min}}$ is safe enough for many patients; hence, dose $x_{1} = x_{\text {min}} + \varepsilon \in (x_{\text {min}},x_{\text {max}})$ with some small value $\varepsilon >0$ can be used as an initial dose for the first patient. The value of $\varepsilon$ may depend on the unit of the dose. The third assumption (A3), called a monotonic dose-toxicity assumption, is the basic principle underlying cytotoxic anticancer agents or a combination of a biologic with a cytotoxic drug being developed for cancer chemotherapy. The maximum dose $x_{\text {max}}$ in the fourth assumption (A4) is the supreme dose of the range, and dose $x_{\text {max}} - \varepsilon \in (x_{\text {min}},x_{\text {max}})$ with some small value $\varepsilon >0$ can be explored as the highest dose in phase I studies. Similar to the first assumption, the fifth assumption (A5) means that the interval $(x_{\text {max}},\infty )$ is out of the range of the studies.

### The maximum tolerated dose

In this subsection, we aim to mathematically define MTD and DLT based on a continuous toxicity response *Y*(*x*) assuming (A1)–(A5). This inevitably requires some probabilistic statements. The definition of MTD based on continuous toxicity response was first conceptualized by authors [34], and recently, Lee et al. [16] modernized the definition for a more practical phase I clinical trial, accommodating multiple adverse events in determining MTD.

For cytotoxic anticancer agents, one fundamental assumption is usually made: the probability of a toxicity response increases with dose. (This is associated with the third assumption (A3) of *Y*(*x*).) A mathematical description of this assumption is as follows. We assume that clinicians have some prior knowledge about a threshold value $\eta > 0$, such that any value *Y*(*x*) greater than $\eta$ may cause the occurrence of DLT in patients assigned with dose *x*. Intuitively, what clinicians want to control is the “probability” of the occurrence of DLT at dose *x*, denoted as $\textbf{Pr}[Y(X) \ge \eta | X = x]$. Clinically, this probability represents the proportion of patients who experience DLT at dose *x*. With these formulations, the fundamental assumption implies that the probability $\textbf{Pr}[Y(X) \ge \eta | X = x]$ monotonically increases with dose *x*. For the safety of patients, in most cases, this probability is controlled by upper-bounding it, so that the number of patients with DLT can be probabilistically restricted. The following defines the MTD:

#### Definition 1

Given prespecified values $\eta >0$, $\gamma \in (0.5, 1)$, and an open interval $(x_{\text {min}}, x_{\text {max}})$ $\subset (0,\infty )$, the MTD is defined to be the largest value of *x* which satisfies the following inequalities

1 $$\begin{aligned} \textbf{Pr}[Y(X)< \eta | X = x] \ge \gamma \quad \text {and}\quad x_{\text {min}}< x < x_{\text {max}}. \end{aligned}$$

Throughout the paper, we shall denote the MTD as $\xi$. Then, the MTD $\xi$ based on Definition 1 can be re-written as follows:

2 $$\begin{aligned} \xi = \text {argsup}_{x\in (x_{\text {min}},x_{\text {max}})} \{x \in \mathbb {R} \mid \textbf{Pr}[Y(X) \ge \eta | X = x] \le 1 -\gamma \}. \end{aligned}$$

Here, the variables $\eta > 0$, $\gamma \in (0.5, 1)$, and $(x_{\text {min}},x_{\text {max}})$ are pre-specified by clinicians at the planning stage of the design.

The medical interpretations of the variables are as follows:

- Maximum toxicity level $\eta >0$: the toxicity level that defines the DLT (i.e., $Y(X) \ge \eta$) and non-DLT (i.e., $Y(X) < \eta$). The determination of the threshold value $\eta$ depends on the specific applications.
- Homogeneity constant $\gamma \in (0.5,1)$: the degree of clinicians’ prior belief in the homogeneity of patients’ toxic reaction against doses to be assigned during phase I clinical trials. A higher value of $\gamma$ implies a stronger homogeneity of the toxic reactions of patients.
- Dose range $(x_{\text {min}},x_{\text {max}}) \subset (0,\infty )$: The minimum dose $x_{\text {min}}$ and the maximum dose $x_{\text {max}}$. These are typically inferred from pre-clinical studies.

We detail some modeling considerations concerning the variables. The clinical meaning of the maximum toxicity level $\eta$ depends on the context of the applications using the continuous toxicity response *Y*(*x*). For example, if *Y*(*x*) is based on a continuous toxicity score as discussed by [16], then $\eta$ represents the maximum toxicity score. If *Y*(*x*) represents the pharmacokinetic exposure of a new drug, then $\eta$ is associated with the maximum tolerated concentration [18, 41].

On the other hand, the homogeneity constant $\gamma$ may have a closer relationship with the characteristics of patients undergoing treatment with a specific agent. Specifically, it indicates whether these patients exhibit homogeneity or heterogeneity in their response to a dose *x* of the new agent [16]. Mathematically, as inferred from the inequality (2), the value $\theta = 1-\gamma$ serves as the least upper bound for the probability of DLT occurrences among patients across the dose range $(x_{\text {min}},x_{\text {max}})$. This constant, denoted as $\theta = 1-\gamma$ in [42], is known as the target toxicity level [43]. Clinically, target toxicity level $\theta$ is the target probability of DLT at MTD, representing the acceptable likelihood of a patient experiencing a DLT at MTD [42]. Normally, $\theta$ is set relatively high when the DLT is reversible or nonfatal condition, and low if it is life-threatening [40]. As a default value, we recommend $\theta = 0.01$ (or equivalently, $\gamma = 0.99$) when dealing with a completely novel agent [16, 34]. With this choice, the safety of patients is prioritized, allowing at most one out of a hundred patients to exhibit DLT. If the agent under consideration has been used before but with variations in schedule, route of administration, or concomitant drugs, the default value for $\gamma$ is often not explicitly defined. Its determination becomes highly dependent on the specific therapeutic area. Some authors suggest that $\gamma$ typically falls within the range of 0.6 to 0.9 (or equivalently, $\theta$ ranging from 0.1 to 0.4). For more details, refer to the Chapter on Phase I Trials in [44], or page 40 in [45].

The dose range $(x_{\text {min}},x_{\text {max}})$ depends on the unit of the drug (e.g., gram, milligram, or microgram) and is mostly inferred from animal studies, meta-analysis, or previous clinical studies with similar drug molecules, etc.

**Fig. 1:**
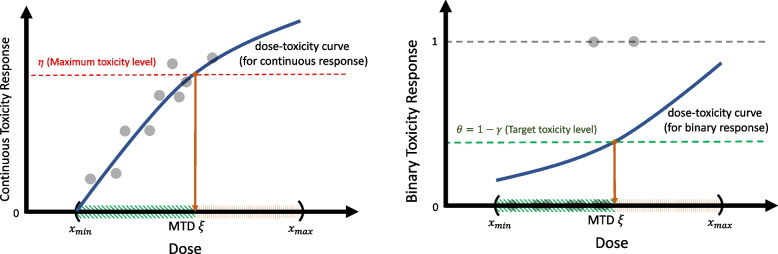
Pictorial illustration of determining an MTD from two different perspectives: continuous toxicity response (left panel) and binary toxicity response (right panel)

Figure 1 provides a visual depiction of the MTD $\xi$ (2) from two distinct perspectives of toxicity outcomes: continuous (left panel) and binary (right panel). In both panels, the *x*-axis represents the dose *x*, while the *y*-axis corresponds to the continuous and binary toxicity responses in the left and right panels, respectively. The binary responses are obtained by dichotomizing the continuous responses using the threshold value of the maximum toxicity level $\eta$. On the left panel, the curve illustrates the dose-toxicity relationship, represented by a monotonic function. The right panel presents the probability of DLT at a given dose *x*, denoted as $\textbf{Pr}[Y(X) \ge \eta | X = x]$ in Eq. (1). In reality, the precise shapes of these curves remain unknown. However, a crucial consideration in phase I cancer studies is that, due to the assumption of a monotonic relationship for anti-cancer drugs, the true curves are expected to monotonically increase over the dose range $(x_{\text {min}},x_{\text {max}})$. The green-shaded intervals represents safe doses that fulfill the condition on the right-hand side of Eq. (2). In contrast, the red intervals consists of highly toxic doses that could potentially induce DLT in patients. Ultimately, the primary objective of phase I clinical trials is to estimate the MTD $\xi$, which corresponds to the upper limit of the green interval.

### A fully sequential adaptive design

The present subsection aims to provide an algorithmic description of a framework of adaptive dose-finding design for finding MTD $\xi$ (2). We explain fully sequential adaptive design (FSAD), which is the default setting in this paper. FSAD is characterized as follows:

- 1. Patients are introduced to the trials individually and sequentially.
- 2. Each patient is assigned an optimal estimate of MTD $\xi$ (2) based on the accumulated patients’ information at interim.

To operate FSAD, some essential ingredients are needed. Suppose that we have a total of *N* patients who can participate in a phase I clinical trial. Let $(x_i, y_i)$ denote the ordered pair (dose, continuous toxicity response against the dose) for the *i*-th patient ($i=1,\cdots ,N$). Let $\mathcal {F}_n = \{(x_i, y_i) \}_{i=1}^{n}$ represent the accrued information from *n* patients ($n=1,\cdots ,N$). Due to the accumulation of patients’ information during the trial, it holds that $\mathcal {F}_1 \subset \mathcal {F}_2 \subset \cdots \subset \mathcal {F}_N$, where the notation $\subset$ represents the subset relationship.

Finally, we need a dose-finding rule (also called a design adaptation rule [4]), which is defined as a mapping from the information space $\varvec{\mathcal {F}}$ to the dose range $(x_{\text {min}}, x_{\text {max}})$:

3 $$\begin{aligned} \mathcal {D} (\cdot ) : \varvec{\mathcal {F}} \longrightarrow (x_{\text {min}}, x_{\text {max}}). \end{aligned}$$

Dose-finding rule $\mathcal {D} (\cdot )$ (3) receives the cumulative information set $\mathcal {F}_n\in \varvec{\mathcal {F}}$ from the first *n* patients as input, and prints out the dose $x_{n+1} = \mathcal {D}(\mathcal {F}_n)$ for the $(n+1)$-th patient that belongs to the interval $(x_{\text {min}} , x_{\text {max}})$. The output $x_{n+1}$ is an optimal dose for the $(n+1)$-th patient. Generally, good operating characteristics of adaptive designs is determined by dose-finding rule $\mathcal {D} (\cdot )$ (3), which is the drive engine of adaptive clinical trial designs.

**Fig. 2:**
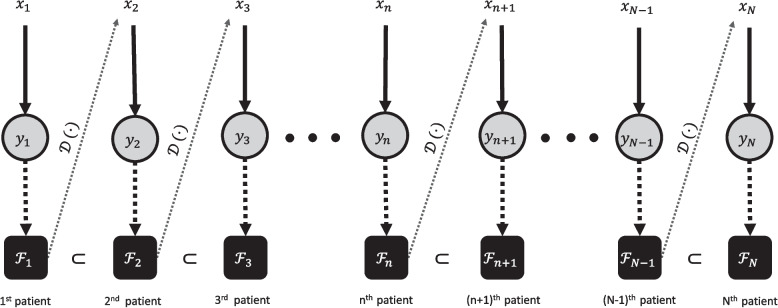
Pictorial description of the fully sequential adaptive design (Algorithm 1). $x_{n}$ represents the dose for the *n*-th patient, $y_{n}$ is the continuous toxicity response for the *n*-th patient, $\mathcal {F}_n = \{(x_i, y_i) \}_{i=1}^{n}$ represents the cumulative information up to *n* patients, and $\mathcal {D} (\cdot )$ is the dose-finding rule. Due to the accumulation of patient information, the subset relationship holds: $\mathcal {F}_1 \subset \mathcal {F}_2 \subset \cdots \subset \mathcal {F}_N$

Algorithm 1 describes four steps to implement FSAD. A pictorial description is displayed in Fig. 2. Starting from the initial dose $x_1 \in (x_{\text {min}}, x_{\text {max}})$, we observe the continuous toxicity response $y_1 = Y(x_{1})$ for the first patient, which then forms the information set $\mathcal {F}_1=\{(x_1,y_1) \}$. Based on $\mathcal {F}_1$, we select an optimal dose for the second patient by $x_2 = \mathcal {D}(\mathcal {F}_{1})$, completing the first cycle. The second cycle starts by introducing the dose $x_2$ to the second patient, and we record his or her response $y_2$, leading to the accrued information $\mathcal {F}_2=\{(x_1,y_1),(x_2,y_2)\}$. We then find an optimal dose for the third patient, that is, $x_3 = \mathcal {D}(\mathcal {F}_{2})$. This cycle continues until we reach the *N*-th patient, who will be assigned the dose $x_N = \mathcal {D}_{\alpha }(\mathcal {F}_{N-1})$ based on the accrued information $\mathcal {F}_{N-1} = \{(x_i, y_i) \}_{i=1}^{N-1}$. The final estimate $x_N$ is then regarded as an optimal estimate of the MTD $\xi$ (2).

**Figure:**
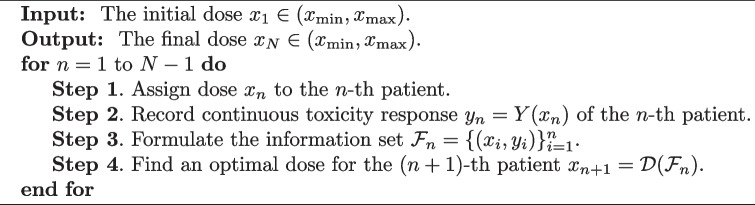
**Algorithm 1** Fully sequential adaptive design

Normally, for safety, the initial dose $x_{1}$ is set to a dose very close to the minimum dose $x_{\text {min}}$ (i.e., $x_{1} = x_{\text {min}} + \epsilon$ with sufficiently small $\epsilon >0$), and we expect that the sequence of doses $(x_{n})$ slowly converges to the targeted MTD $\xi$ (2) as the trials progress. Here, the dose sequence $(x_{n})$ does *not* need to be monotonic (that is, the inequalities $x_{1} \le x_{2} \le \cdots \le x_{N}$ are not required) because we may de-escalate the dose if some previous doses are overly toxic.

In practice, patients are often treated in a cohort size of three. In this case, FSAD (Algorithm 1) can be can be easily generalized to cohort sequential adaptive design (CSAD). Suppose that there are in total *N* cohort groups and patients are enrolled in a cohort of size of *C*, where *C* is a fixed positive integer (if $C = 1$, then CSAD reduces to FSAD; $C = 3$ is often used for CSAD). Therefore, we have in total $N\cdot C$ patients who can participate in a phase I clinical trial. Algorithm for CSAD can be obtained by (1) assigning dose $x_{n}$ to *C* patients in the *n*-th cohort; (2) recording *C*-dimensional vector of continuous toxicity responses $y_{n,1:C}=(y_{n1},\cdots ,y_{nC})$ from *C* patients in the *n*-th cohort; (3) formulating the information set $\mathcal {F}_N = \{(x_i, y_{i,1:C}) \}_{i=1}^{N}$; and (4) finding an optimal dose for the $(n+1)$-th cohort based on the accumulated cohorts’ information at interim by $x_{n+1} = \mathcal {D} (\mathcal {F}_n)$.

### Diagnosis of a dose-finding rule

Now, the fundamental question is this: given a dose-finding rule $\mathcal {D} (\cdot )$ (3) under FSAD (Algorithm 1), how can we evaluate the algorithm’s utility for actual phase I clinical trials? Due to the small-sample nature of the problem, perhaps the best approach is to examine this through conducting clinical trial simulations as follows. The basic idea here is to assess the “self-sequential learning ability” by replicating a large number of phase I clinical trials and observing their clinical operating characteristics. To achieve this, we begin by specifying three variables $(N, x_{1}, \xi _{0})$ as follows: setting the sample size *N* to be small enough (e.g., $N=20$ or 25 patients); determining the initial dose $x_{1}$ to be sufficiently close to the minimum dose $x_{\text {min}}$; and fixing the MTD to a “true” value denoted as $\xi _{0}$ (2). The subscript “0” is used to indicate the “truth.”

Next, we define a data generating distribution for simulating toxicity responses $y_{n}$ given doses $x_{n}$ ($n=1,\cdots ,N$). Conceptually, this distribution takes the form of a conditional distribution, denoted as $P_{\xi }(y|x)$, parameterized by the MTD $\xi \in (x_{\text {min}} , x_{\text {max}})$. With this assumption, we can implement Step 2 in Algorithm 1 by recording the response $y_{n} \sim P_{\xi _{0}}(y|x_{n})$ ($n=1,\cdots ,N$). Here, we assume that the *N* responses ($y_{1},\cdots ,y_{N}$) are conditionally independent given the parameter $\xi$ and covariates ($x_{1},\cdots ,x_{N}$). Steps 3 and 4 in Algorithm 1 remain unchanged. These modifications to Algorithm 1 operate on a single simulated phase I clinical trial, and the outcome will be the information set $\mathcal {F}_N = \{(x_i, y_i) \}_{i=1}^{N}$ obtained from *N* patients. The dose assigned to the last patient, $x_N$, is then considered the final estimate of the MTD.

The distribution $P_{\xi }(y|x)$ describes the relationship between toxicity and dose. In most model-based designs, the underlying distribution $P_{\xi }(y|x)$ is assumed to be parsimonious; otherwise, the doses for the first few patients could be suboptimal due to the small sample size. This may violate *individual ethics*—doing what is best for current patients in the trial. For that reason, the Bernoulli distribution is conventionally used if *y* represents a binary toxicity response [3, 26, 27], and the Gaussian distribution is often used if *y* is a continuous toxicity response [16, 34], while allowing only few number of parameters to describe some dynamics (e.g., slope, intercept, curvature, interpatient variability, etc.) of the dose-toxicity relationship. Therefore, one may rewrite the distribution $P_{\xi }(y|x)$ more concretely as $P_{(\xi ,\phi )}(y|x)$, where $\phi$ represents some statistical nuisance parameter(s) describing such dynamics. However, for readability, we will keep using the notation $P_{\xi }(y|x)$ until this subsection.

The eventual success of model-based dose-finding design relies on a dose-finding rule $\mathcal {D}(\cdot )$ (3) that enables the design to generate the sequence of doses $(x_{n})$ by cycling through the steps in Algorithm 1. We hope that the sequence $(x_{n})$ converges to the true MTD $\xi _{0}$ as *n* grows. Inappropriate choices of the dose-finding rule will lead to very slow convergence of the sequence or, in the worst scenario, it will never converge to the truth.

With c*n*-th patient, $\mathcal {F}_n = \{(x_i, y_i) \}_{i=1}^{n}$, we can generate the following four sequences of performance metrics to assess the utility of a proposed dose-finding rule $\mathcal {D} (\cdot ): \varvec{\mathcal {F}} \longrightarrow (x_{\text {min}} , x_{\text {max}})$:

- Number of patients with DLT (NPD): $\text {NPD}(n) = \sum _{i=1}^{n} {\textbf {1}}( y_{i} \ge \eta )$.
- Number of patients overdosed (NPO): $\text {NPO}(n) =\sum _{i=1}^{n} {\textbf {1}}(x_{i}>\xi _{0})$.
- Bias to MTD (BTM): $\text {BTM}(n) =x_{n} - \xi _{0}$.
- Square root of the relative mean squared error (RMSE): $\text {RMSE}(n) = |x_{n} - \xi _{0}|/\xi _{0}$.

The notation ${\textbf {1}}(\cdot )$ represents the indicator function. NPD and NPO are intended to measure the safety of a treatment as drug dose, whereas BTM and RMSE evaluate estimation accuracy of a dose-finding rule. Specifically, NPD, NPO, and BTM are metrics tailored to phase I studies, while RMSE is generally reported when the parameter of interest is positive [46]. Generally, smaller values of NPD and NPO indicate better clinical safety. As for BTM, a smaller negative value is desired because we normally expect $x_{n}$ to be smaller than the true MTD $\xi _{0}$ but close enough to $\xi _{0}$. Lastly, the smaller the square root of the RMSE, the better the estimation accuracy. (In this paper, we will simply denote the metric as “RMSE” instead of “SRMSE” to avoid lengthy notation.)

By evaluating the four metrics - $\text {NPD}(n)$, $\text {NPO}(n)$, $\text {BTM}(n)$, and $\text {RMSE}(n)$ - at each value of $n=1,2,3,\cdots ,\infty$, we can access asymptotic aspects of a dose-finding rule $\mathcal {D} (\cdot )$. According to the law of large numbers, $\text {NPD}(n)/n$ and $\text {NPO}(n)/n$ converge almost surely to the probability of DLT (that is, $\textbf{Pr}[Y > \eta ]$) and the probability of overdosing event (that is, $\textbf{Pr}[X > \xi _{0}]$) as *n* goes to infinity, respectively. By using the law of total expectation, we have $\textbf{Pr}[Y> \eta ] = \mathbb {E}[{\textbf {1}}(Y> \eta )]= \mathbb {E}[\mathbb {E}[{\textbf {1}}(Y(X)> \eta )|X]] = \mathbb {E}[\textbf{Pr}[Y(X) > \eta ]|X]] \le \mathbb {E}[1 -\gamma |X] =1 -\gamma$. Therefore, it holds $\text {lim}_{n \rightarrow \infty } \text {NPD}(n)/n \le 1 -\gamma$ almost surely. This implies that the proportion of patients with DLT is asymptotically controlled by the homogeneity constant $\gamma$, or equivalently, target toxicity level $1-\gamma$. For example, by specifying $\gamma = 0.9$, maximally one patient out of ten patients may show DLT in the long run. Later, we will see that the probability of overdosing event, $\textbf{Pr}[X > \xi ]$, can be also controlled by clinicians by using “feasibility bound,” but unlike the controlling mechanism of the probability of DLT, $\textbf{Pr}[Y > \eta ]$, done by $\gamma$, the probability of overdosing event can be controlled in a Bayesian way. As for the two accuracy metrics, the sequences $(\text {BTM}(n))$ and $(\text {RMSE}(n))$ should converge to 0 as *n* grows; a dose-finding rule with this property is said to have a consistency property [34].

Due to the small sample nature of phase I studies, a practically useful diagnosis of a dose-finding rule $\mathcal {D} (\cdot )$ can be performed by cross-sectional analyses of the four metrics evaluated at a certain number $n=N$, where *N* is a small natural number, say $15-30$, and by checking the metrics via replicated numerical experiments under diverse scenarios by varying design parameters $\gamma$, $\eta$, and $(x_{\text {min}},x_{\text {max}})$ that were used in defining the MTD and DLT. This way, we can assess whether the rule $\mathcal {D} (\cdot )$ is robust enough to handle variations in environmental variables and can be used in real phase I studies across various therapeutic areas. Particularly, a dose-finding rule $\mathcal {D} (\cdot )$ with a positive BTM (that is, $x_{N} > \xi _{0}$) is inappropriate for actual phase I clinical trials because it is highly likely that overly toxic doses would be suggested by the underlying model.

### Non-linear dose-toxicity curve

Let *Y*(*x*) denote continuous toxicity response, assuming the validity of five assumptions (A1)–(A5). Additionally, we assume that the patients’ responses at the minimum dose $x_{\text {min}}$ have been appropriately transformed and centered around zero, ensuring $\mathbb {E}[Y(x)|x = x_{\text {min}}]=0$. In this paper, we further assume a non-linear correlation between toxicity and dose, expressed as follows:

4 $$\begin{aligned} y = \beta (x - x_{\text {min}})^{\nu } + \sigma \epsilon , \quad \epsilon \sim \mathcal {N}(0,1), \end{aligned}$$

where $\beta$, $\nu$, and $\sigma$ are positive real numbers. Here, $\mathcal {N}(\mu ,\sigma ^{2})$ denotes the Gaussian distribution with mean $\mu$ and standard deviation $\sigma$. The parameters in the non-linear Eq. (4) have specific interpretations. Specifically, $\beta$ represents the slope of the dose-toxicity curve, and when $\nu =1$, it represents the rate of the increment of toxicity response per unit of dose. On the other hand, $\nu$ represents the non-linearity parameter, with a higher value indicating a greater curvature of the dose-toxicity curve. Finally, $\sigma$ represents the standard deviation of the toxic reaction of patients at a given dose *x*, describing inter-patient variability.

Note that the non-linear regression (4) reduces to a simple linear regression when $\nu = 1$. This simple linear regression was studied by [16, 34] for the application of phase I clinical trials. Specifically, Eichhorn and Zacks [34] discussed a Bayesian estimation of the slope parameter $\beta$, while fixing the standard deviation $\sigma$. On the other hand, Lee et al. [16] aimed to estimate both parameters in a fully Bayesian way. Estimating $\sigma$ is crucial because phase I cancer trials might enroll terminal cancer patients with different types of malignant tumors at various disease stages; hence, the patient population is usually heterogeneous [47]. Both of the previous studies assumed that the toxicity response is linear in dose, lacking the flexibility to describe the toxicity-dose curve when the true curve is non-linear. In this paper, we extend the previous research by introducing the non-linear parameter $\nu$ and estimating it in a fully Bayesian way.

**Fig. 3:**
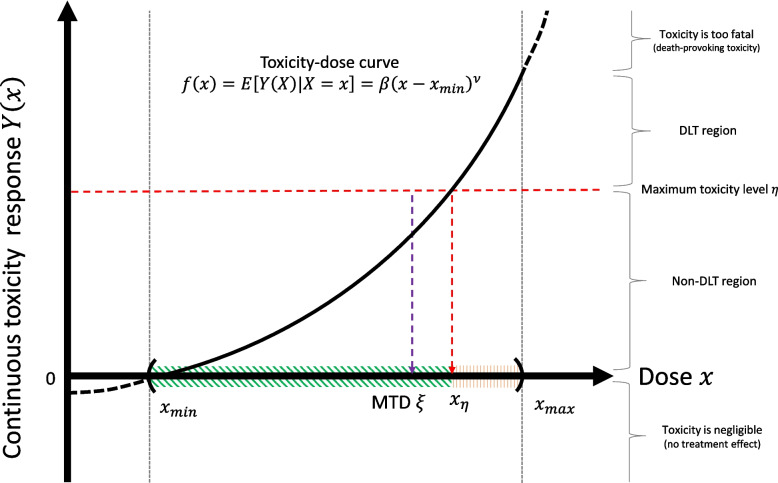
Pictorial description of the non-linear dose-toxicity curve

A closed-form expression of MTD $\xi$ (2) can be derived by providing some constraints on the parameters $(\beta ,\nu ,\sigma )$:

#### Theorem 1

Consider a non-linear regression to describe the relationship between toxicity and dose: $y \sim \mathcal {N}(\beta (x - x_{\text {min}})^{\nu },\sigma ^{2})$. Suppose that the slope and non-linearity parameters and standard deviation are restricted by $\beta > (\eta - \sigma \Phi ^{-1}(\gamma ))/(x_{\text {max}} - x_{\text {min}})^{\nu }$, $\nu > 0$, and $0< \sigma < \eta /\Phi ^{-1}(\gamma )$. Then, MTD $\xi$ (2) is given by

5 $$\begin{aligned} \xi = \xi (\beta ,\nu , \sigma ) = x_{\text {min}} + \left( \frac{\eta - \sigma \Phi ^{-1}(\gamma )}{\beta } \right) ^{1/\nu }. \end{aligned}$$

#### Proof

See the Appendix.  

Figure 3 is a pictorial description of the mean part of the dose-toxicity curve (4), that is, $f(x) = \mathbb {E}[Y(X)|X=x] = \beta (x - x_{\text {min}})^{\nu }$. The curve is convex if $\nu > 1$ (as shown in Fig. 3), line if $\nu = 1$, and concave if $0<\nu <1$. The maximum toxicity level $\eta$ divides the dose-toxicity plane into the DLT region $\{(x,y) | x \in (x_{\text {min}},x_{\text {max}} ), y \ge \eta \}$ and the non-DLT region $\{(x,y) | x \in (x_{\text {min}},x_{\text {max}} ), 0< y < \eta \}$. The two regions, $\{(x,y) | x \in (0, x_{\text {min}}], y \le 0 \}$ and $\{(x,y) | x \in [x_{\text {max}},\infty ), y \ge \beta (x_{\text {max}} - x_{\text {min}} )^{\nu }\}$, represent sub-therapeutic and overly toxic areas, respectively, associated with assumptions (A1) and (A5). Normally, clinicians believe that MTD $\xi$ (5) belongs to the dose range $(x_{\text {min}},x_{\text {max}})$. By using elementary calculus, the solution of the equation $f(x) = \eta$ is $x_{\eta } = x_{\text {min}} + (\eta /\beta )^{1/\nu }$, represented as the red vertical dashed line in the panel. Therefore, MTD $\xi$ (5) is located on the left side of the point $x_{\eta }$ since it always holds $\sigma \Phi ^{-1}(\gamma ) > 0$ and $\gamma \in (0.5,1)$: see the violet vertical dashed line.

### Three-parameter non-linear dose-finder (3PND)

Due to the small-sample, sequential nature of phase I clinical trials, the Bayesian framework is preferred for accurately estimating the MTD $\xi$ (5) than frequentist framework in the literature [4, 48, 49]. From a Bayesian viewpoint, the parameters $\beta$, $\nu$, and $\sigma$ are considered random variables, making MTD $\xi$ a random variable as well. This means that the uncertainty underlying the estimation of the MTD $\xi$ can be probabilistically described once an appropriate prior $\pi (\beta , \nu , \sigma )$ is specified. This advantage of Bayesian methodology in phase I clinical trials does not exist in traditional rule-based designs [50].

We propose a three-parameter non-linear dose-finder (3PND) as a fully Bayesian model-based design. Given the information set $\mathcal {F}_n = \{(x_i, y_i) \}_{i=1}^{n}$, the hierarchy of 3PND is given as follows:

6 $$\begin{aligned} y_{i}|\beta ,\nu , \sigma{} & {} \sim \mathcal {N}\left(\beta (x_{i} - x_{min})^{\nu }, \sigma ^2\right),\quad (i=1,\cdots ,n),\end{aligned}$$

7 $$\begin{aligned} \beta |\nu ,\sigma{} & {} \sim \pi (\beta |\nu ,\sigma ) = \mathcal {U}(l(\sigma ,\nu ),u(\sigma ,\nu )), \end{aligned}$$

8 $$\begin{aligned} \sigma{} & {} \sim \pi (\sigma ) = \mathcal {C}^{+}(0,1) \mathcal {I}_{(0,\eta /\Phi ^{-1}(\gamma ))},\end{aligned}$$

9 $$\begin{aligned} \nu{} & {} \sim \pi (\nu ) = log \mathcal {N}(0,\delta ^{2}), \end{aligned}$$

where $l(\sigma ,\nu ) = \{\eta - \sigma \Phi ^{-1}(\gamma )\}/(x_{\text {max}} - x_{\text {min}})^{\nu }$ and $u(\sigma ,\nu ) =\{\eta /(x_{\text {max}} - x_{\text {min}})^{\nu }\} + \sigma \Phi ^{-1}(\gamma )$. $\Phi (z)$ is the cumulative distribution function of the standard normal distribution. Notation $\mathcal {U}(l,u)$ represents the uniform distribution supported on open interval (*l*, *u*). $\mathcal {C}^{+}(0,1) \mathcal {I}_{(0, d)} \propto \{1/(1+z^2) \}\cdot \mathcal {I}_{(0, d)}$ represents the unit scaled half-Cauchy distribution truncated on the interval (0, *d*). $log \mathcal {N}(0,\delta ^{2})$ represents the log-normal distribution with a unit median and a scale $\delta >0$. See the Appendix for the detail of posterior computation.

The prior distribution $\pi (\beta , \nu , \sigma )$ (7)–(8) is conceptually weakly informative, imposing minimal restrictions on the prior, while assuring that the MTD $\xi$ lies in the dose range $(x_{\text {min}}, x_{\text {max}})$ with probability 1. Specifically, the prior is constructed as follows: first, we assume a flat prior for $\beta$ given $\nu$ and $\sigma$ (i.e., $\pi (\beta |\nu , \sigma ) \propto 1$, supported on $(-\infty , \infty )$), a unit-scaled half-Cauchy prior for $\sigma$ (i.e., $\pi (\sigma ) \propto 1/(1+\sigma ^2)$, supported on $(0, \infty )$), and a log-normal prior for $\nu$ (i.e., $\pi (\nu ) = \log \mathcal {N}(0,\delta ^{2})$, supported on $(0, \infty )$), centered around one, with the scale hyper-parameter $\delta > 0$. After that, we restrict the support of the joint prior $\pi (\beta , \nu , \sigma )$ to ensure that the MTD $\xi$ (5) belongs to the dose range $(x_{\text {min}}, x_{\text {max}})$. Note that the uniform and half-Cauchy distributions are considered non-informative and weakly informative priors, respectively. Log-normal distribution is a sub-exponential distribution, a class of heavy-tailed distributions studied by Lee [51] for a small-sample problem. Its tail-heaviness provides the flexibility of non-linearity of the dose-toxicity curve. Default values for $\delta$ are $\delta = 0.1$ or 0.5. In real applications, a plausible value for $\delta$ can be also chosen via sensitivity analysis. The higher the value of $\delta$, the stronger the prior guess on the non-linearity of the dose-toxicity relationship. Under the prior formulation, the values of $\delta$, $\eta$, $\gamma$, $x_{\text {min}}$, and $x_{\text {max}}$, used in defining the MTD (1), are introduced as the hyperparameters of the 3PND.

**Fig. 4:**
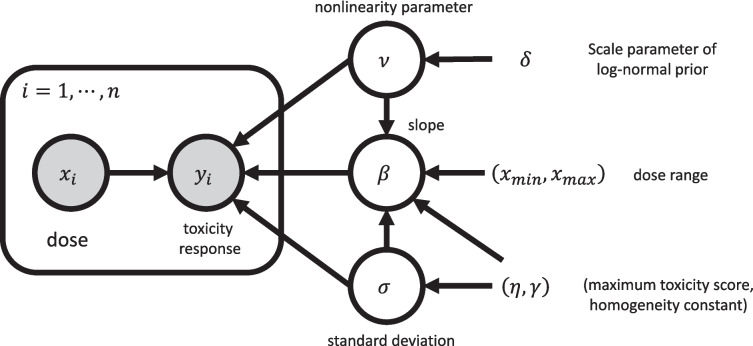
3PND as a graphical model

Figure 4 displays a directed asymmetric graphical (DAG) model representation of the 3PND. Following the grammar of the graphical model (Chapter 8 of [52]), the circled variables indicate stochastic variables, while the observed ones are additionally colored in gray. Non-stochastic quantities are uncircled. The arrows indicate the conditional dependency between the variables.

Some difficulty in prior elicitation is briefly discussed. As noted in the guidance for the use of Bayesian statistics provided by the Food and Drug Administration [53], special care is needed in incorporating appropriate prior information in Bayesian modeling. Specifically, the prior information should allow the Bayesian model to be flexible and efficient in identifying any pattern during trials [9]. This implies that we should prevent a suggested Bayesian model from being overly dominated by the prior information, and to that end, imposing weaker prior information may be more reasonable. However, given the FSAD setting, patients are introduced to the trials sequentially and individually. Hence, allowing too weak prior information may lead to unstable parameter estimations, and it is highly likely that the first few patients are suboptimally dosed. The CSAD setting also suffers from this issue. This may contradict the individual ethics mentioned earlier. Therefore, a good model should retain a reasonable balance between these two competing requirements.

### Theoretical guarantee of MTD estimation using 3PND

The fundamental principle of a dose-finding study design is to allocate each included subject to the current best estimate of MTD. The central question we may ask here is, “Will the proposed dose-finding algorithm discover the true MTD given a large number of patients, such as 100 patients or even more, say, 1,000 patients?” Although enrolling such a significant number of patients is not practical, one might seriously doubt the proposed design if it fails to detect the MTD even with such an extensive sample size.

In the present subsection, we provide a theoretical demonstration that the updated knowledge regarding the MTD, denoted as $\xi$, progressively becomes more accurate and precise as the number of patients increases. This idealistic phenomenon is established through the concept of posterior consistency [54]. To elaborate, let $\xi _0$ represent the true value of MTD $\xi$. Our objective is to establish that, as long as the true value $\xi _0$ resides within the dose range $(x_{\text {min}}, x_{\text {max}})$, the posterior distribution of MTD $\xi$ becomes increasingly concentrated around $\xi _0$ with an expanding sample size. In this case, we say that, “the posterior distribution of MTD $\xi$ is consistent at $\xi _0$” [55]. To establish posterior consistency, we leverage Doob’s theorem [56] to provide a sufficient condition for the posterior consistency of the 3PND. This theorem asserts that “for any prior $\pi$, the posterior is consistent at every value in the parameter space except, possibly, on a set of $\pi$-measure zero.”

#### Theorem 2

Within the hierarchy of the 3PND (6) – (9), the posterior distribution of the MTD $\xi$ is consistent at any value of $\xi _0$ within the dose range $(x_{\text {min}},x_{\text {max}})$.

#### Proof

See the Appendix.  

Theorem 2 guarantees that as long as the dose range $(x_{\text {min}}, x_{\text {max}})$ encompasses the targeted MTD $\xi _0$, the posterior distribution of MTD $\xi$ remains consistent at $\xi _0$. In reality, since the true MTD $\xi _0$ is unknown, it is advisable to select a dose range that is sufficiently broad to encompass the true value $\xi _0$. However, the interval should not be overly wide as it may require a larger sample size than otherwise.

### A dose-finding rule using 3PND

A dose-finding rule $\mathcal {D}(\cdot )$ (3) is the driving engine to operate an adaptive dose-finding design (Algorithm 1). In the present subsection, we derive a dose-finding rule based on the 3PND (6) – (9) to satisfy the desiderata.

Suppose that we have accrued information from *n* patients $\mathcal {F}_n = \{(x_i, y_i)\}_{i=1}^{n}$, which is the input of the mapping $\mathcal {D}(\cdot )$ (3). The goal is now to select a dose for the $(n+1)$-th patient (denoted as $x_{n+1}$), having observed $\mathcal {F}_n$. In the dose assigning procedure, our aim is to select an optimal dose $x_{n+1}$ while controlling the following two quantities:

- (i) The probability of the occurrence of DLT from patients
- (ii) The posterior probability of the event of overdosing to patients

The controlling mechanism of the probability of DLT has already been taken into consideration in the formula of MTD $\xi$ (5), which can be controlled by changing the homogeneity constant $\gamma \in (0.5,1)$ (see Fig. 1). Noting from Eq. (2), $1 - \gamma$ represents the maximum proportion of patients with DLT at MTD. A reasonable value for $\gamma$ may depend on the specific therapeutic area. If the anti-cancer agent is entirely novel, a recommended value for $\gamma$ is 0.99 so that probabilistically, at most one patient out of a hundred patients shows the DLT status. Otherwise, the default value for $\gamma$ is often not explicitly defined, as it highly depends on a certain therapeutic area. For example, values may range from 0.6 to 0.9.

On the other hand, to control the posterior probability of the overdosing event, we introduce a new variable, called (Bayesian) feasibility bound denoted as $\alpha$ in the literature [16, 34]. To describe the concept, let us assume that $\Pi _n(x) = \textbf{Pr}[\xi \le x | \mathcal {F}_n]$ denotes the posterior cumulative distribution function of MTD $\xi$ given $\mathcal {F}_n$. With a value $\alpha \in (0,1)$ set by clinicians, the selected dose $x_{n+1}$ is the posterior $\alpha$-quartile for the MTD $\xi$:

10 $$\begin{aligned} \Pi _n(x_{n+1}) = \textbf{Pr}[\xi \le x_{n+1} | \mathcal {F}_n] \le \alpha . \end{aligned}$$

Inequality (10) implies that the posterior probability of the event of overdosing to the $(n+1)$-th patient is bounded above by the constant $\alpha$. It is important to note that, unlike the homogeneity constant $\gamma$, the feasibility bound $\alpha$ is neither a hyper-parameter nor a variable introduced to define MTD and DLT. Essentially, $\alpha$ is a pure Bayesian apparatus to control the convergence rate of the dose sequence $(x_{n})$ toward the MTD. This quantile-based dose selection scheme has also been adopted in EWOC [40] and its extension [15].

In practice, the proper value of $\alpha$ can be chosen through extensive simulations, depending on factors such as the disease, patients’ characteristics, and the number of patients to be enrolled. A lower value for $\alpha$ leads to a more conservative dose escalation, allowing for a smaller jump size from the current dose $x_n$ to the next dose $x_{n+1}$ during the trials. On the other hand, a higher value for $\alpha$ results in a more aggressive jump size from $x_n$ to $x_{n+1}$. We recommend using $\alpha = 0.001$, 0.01, 0.05, or 0.1 as default values of the feasibility bound. Sometimes, we can use a variable feasibility bound $\alpha$, starting with some small value of $\alpha$, and as the trial progresses, $\alpha$ increases in small increments [42]. In practice, an optimal value for the $\alpha$ can be determined by extensive simulation experiments by trying different value of $\alpha$, given values of $\gamma$ and sample size *N*.

Now, we describe how to obtain $x_{n+1}$ which satisfies the inequality (10). Because $\xi$ is a continuous random variable, we have the theoretical expression $x_{n+1} = \Pi _{n}^{-1}(\alpha )$, where $\Pi _{n}^{-1}(\cdot )$ is the inverse function of $\Pi _{n}(\cdot )$. However, the function $\Pi _n(\cdot )$ is not known in a closed-form distribution; hence, it is difficult to obtain $x_{n+1}$ in a closed-form solution. Instead, we resort to a Markov chain Monte Carlo (MCMC) algorithm [57] to approximate $x_{n+1}$. Algorithm 2 describes the steps to produce the next dose $x_{n+1}$ based on accrued patients’ information $\mathcal {F}_{n}$ and feasibility bound $\alpha$. More technically, the next dose $x_{n+1}$ is an output of the function $\mathcal {D}_{\alpha }(\cdot )$ evaluated at the input $\mathcal {F}_{n}$: that is, $x_{n+1} = \mathcal {D}_{\alpha }(\mathcal {F}_{n}) \in (x_{\text {min}}, x_{\text {max}})$ $(n=1,2, \cdots , N-1)$. In the notation $\mathcal {D}_{\alpha } (\cdot )$, the Greek letter $\alpha$ is subscripted to emphasize that $\alpha$ is fixed during trials.

**Figure:**
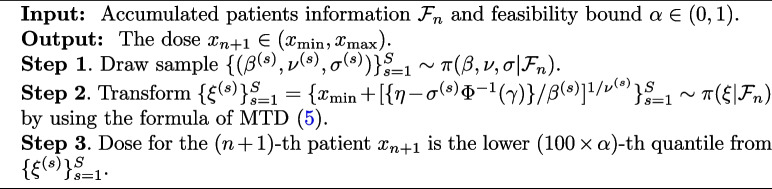
**Algorithm 2** Dose-finding rule $\mathcal {D}_{\alpha } (\cdot ) : \varvec{\mathcal {F}} \longrightarrow (x_{\text {min}} , x_{\text {max}})$

Algorithm 2 represents the vanilla version, where the dose-searching process relies solely on the quantile Eq. (10). The development of an efficient sampling algorithm in Step 1 of Algorithm 2 is pivotal to the success of the dose-finding algorithm. We have devised a sampling algorithm that combines the Gibbs sampler [58], the slice sampler [59], and the elliptical slice sampler [60]. Detailed information can be found in the Appendix.

## Results

To assess the operating characteristics and utility of 3PND in phase I cancer clinical trials, in this section, we conduct extensive simulation experiments and apply the proposed design to an optimal dose-finding problem using a dose-response dataset resampled from Friedman et al. [38].

### Simulation experiments

#### Outline of simulation experiments

To assess the method’s average behavior, we perform a simulation study. The general setup adopted here is similarly designed to the simulation experiments from [16, 34]. As we are mainly interested in evaluating the operating characteristics of the design, experiments are conducted based on the vanilla dose-finding algorithm described in Algorithm 2.

We compare four dose-finding algorithms that mainly differ in the enrollment schedule of patients with the same number of total patients. Additionally, we want to explore the “Exploration-Exploitation Dilemma” [61]. As normally encountered in many sequential learning problems, there is a trade-off between the exploration of new knowledge about MTD and the exploitation of old knowledge assuring patients’ safety. Optimal performance usually requires some balance between exploratory and exploitative behaviors. One of the benefits of the dose-response modeling framework suggested in this paper is that this balance can be controlled by clinicians. We demonstrate this via simulation experiments.

The four algorithms are denoted as (1) FSAD (acc); (2) FSAD (std); (3) CSAD (acc); and (4) CSAD (std). The first two algorithms are fully sequential adaptive designs with accelerated and standard dosing strategies, respectively, whereas the third and fourth ones are cohort sequential adaptive designs with a cohort size of three $(C=3)$, with accelerated and standard dosing strategies, respectively.

#### Simulation set-up

To simulate a phase I clinical trial, experimenter needs to specify the following five categories of variables: (1) the total number of patients *N*; (2) the maximum toxicity level $\eta$, the homogeneity constant $\gamma$, and the dose range $(x_{\text {min}},x_{\text {max}})$; (3) the feasibility bound $\alpha$; (4) the initial dose $x_1$ for the first patient; and (5) true values of MTD $\xi _{0}$, standard deviation $\sigma _{0}$, and non-linearity parameter $\nu _{0}$. Note that once the values of $\eta$, $\gamma$, $x_{\text {min}}$, $\xi _{0}$, $\sigma _{0}$, and $\nu _{0}$ are determined, then the true value of the slope parameter $\beta _{0}$ is automatically derived through the formula (5), that is, $\beta _{0} = (\eta - \sigma _{0} \Phi ^{-1}(\gamma ))/(\xi _{0} - x_{\text {min}})^{\nu _{0}}$.

After specifying the aforementioned variables, we generate a continuous toxicity response $y_{i} = Y(x_{i})$ according to the dose-toxicity curve (4), that is, $y_{i} \sim P_{(\xi _{0},\nu _{0},\sigma _{0})}(y|x_{i}) = \mathcal {N}(y|\beta _{0} (x_{i} - x_{\text {min}})^{\nu _{0}}, \sigma _{0}^{2})$, and follow the procedure described the previous section to examine the operating characteristics of dose-finding algorithms. Provided a sample size *N*, the eventual outcome based on FSAD (and similarly for CSAD) is the sequence of doses $(x_{1},x_2, \cdots ,$ $x_n, \cdots , x_N)$, and we evaluate the four metrics, $\text {NPD}(N)$, $\text {NPO}(N)$, $\text {BTM}(N)$, and $\text {RMSE}(N)$.

We assume that the scale of the continuous toxicity response $y =Y(x)$ is aligned with the toxicity grade information in CTCAE with adjustment as similarly done by [16]. More specifically, on average, the values $y = 0, 1,2,3$, and 4 indicate mild, moderate, severe, life-threatening, and death-related toxicity of an adverse event against a dose *x*. These values are corresponding to CTCAE Grade 1, 2, 3, 4, and 5, respectively; see [37] for more detail about the generic symptom of adverse events. To check small-sample performance, we set the number of total patients to be $N=18$, 30, and 45, and replicate 1000 times. Eventually, we report median values of the four metrics obtained from replications.

We experiment with two dosing strategies, accelerated and standard dosing strategies. To that end, we set the values of the homogeneity constant $\gamma$ and the feasibility bound $\alpha$ accordingly while fixing other variables. Accelerated design is more inclined to produce a fast and accurate estimation of MTD with a higher risk of DLT and overdosing, and standard design indicates the the acceleration is not used.

To summarize, the following are the variables we set for the simulation experiment:

- Number of patients: $N\in \{18, 30, 45\}$.
- Variables specific to accelerated designs: $\gamma =0.9$ and $\alpha = 0.01$.
- Variables specific to standard designs: $\gamma =0.99$ and $\alpha = 0.001$.
- Variables describing the degree of non-linearity of dose-toxicity curve: $\nu _{0}\in \{0.6, 1, 1.4\}$.
- Variables shared in accelerated and standard designs: $\delta = 0.1$, $\eta =3$, $(x_{\text {min}},x_{\text {max}})=(5,80)$, $x_{1}=6$, $\xi _{0}=50$, and $\sigma _{0}=0.1$.

Under the above specification, a patient shows DLT if the patient’s toxicity response *y* is greater than $\eta =3$ (that corresponds to CTCAE Grade 4), and a patient is overdosed if assigned dose *x* is greater than the true MTD $\xi _{0}$.

#### Simulation results

Table 1 presents the results of simulation experiments based on the four designs. The second column in the table indicates the curvature of the true dose-toxicity curve: linear ($\nu _{0}=1$), strictly convex ($\nu _{0}=1.4$), and strictly concave ($\nu _{0}=0.6$). The best-performing algorithm for each metric is highlighted in bold within each row. Overall, all designs exhibit excellent consistency in MTD estimation across various sample sizes and curvatures, as indicated by the presence of small negative values for BTMs alongside small positive values for RMSEs. It is noteworthy that accuracy improves with increasing sample size, leading to smaller RMSEs as *N* increases. This consistency is theoretically guaranteed, as demonstrated in Theorem 2.

**Table 1:** Results of simulation studies

	FSAD (acc)				FSAD (std)				CSAD (acc)				CSAD (std)			
Curvature	NPD	NP0	BTM	RMSE	NPD	NP0	BTM	RMSE	NPD	NP0	BTM	RMSE	NPD	NP0	BTM	RMSE
*N*=18																
Linear	**0**	**0**	**−1.46**	**0.03**	**0**	**0**	−2.63	0.05	**0**	**0**	−1.59	**0.03**	**0**	**0**	−2.8	0.06
Convex	**0**	1	**−1.24 **	**0.02**	**0**	**0**	−2.25	0.05	3	3	−1.42	0.03	**0**	**0**	−2.31	0.05
Concave	**0**	**0**	** −4.9 **	**0.1**	**0**	**0**	−8.23	0.16	**0**	**0**	−8.47	0.17	**0**	**0**	−13.19	0.26
*N*=30																
Linear	1	**0**	** −1.1 **	**0.02**	**0**	**0**	−1.94	0.04	**0**	**0**	−1.12	**0.02**	**0**	**0**	−1.92	0.04
Convex	1	1	** −0.84 **	**0.02**	**0**	**0**	−1.53	0.03	3	3	−0.98	**0.02**	**0**	**0**	−1.59	0.03
Concave	**0**	**0**	** −2.65 **	**0.05**	**0**	**0**	−4.54	0.09	**0**	**0**	−3.03	0.06	**0**	**0**	−5.02	0.1
*N*=45																
Linear	1	**0**	−0.85	**0.02**	**0**	**0**	−1.46	0.03	1	**0**	** −0.83 **	**0.02**	**0**	**0**	−1.45	0.03
Convex	1	1	** −0.69 **	**0.01**	**0**	**0**	−1.21	0.02	3	3	−0.74	**0.01**	**0**	**0**	−1.19	0.02
Concave	**0**	**0**	−1.89	**0.04**	**0**	**0**	−2.96	0.06	**0**	**0**	** −1.79 **	**0.04**	**0**	**0**	−3.04	0.06

Note: The best-performing algorithm for each metric is highlighted in bold within each row. FSAD (acc), FSAD (std), CSAD (acc), and CSAD (std) correspond to dose-finding algorithms based on 3PND, using accelerated fully sequential design, standard fully sequential design, accelerated cohort sequential design, and standard cohort sequential design, respectively. The second column indicates the curvature of the true dose-toxicity curve: linear ($\nu _{0}=1$), strictly convex ($\nu _{0}=1.4$), and strictly concave ($\nu _{0}=0.6$)

Regarding safety, the standard designs provide a safer dosing strategy in terms of NPD and NPO when compared to the accelerated designs. Notably, all standard designs yield a median value of zero for both NPD and NPO. In terms of the accuracy of MTD estimation, the accelerated designs outperform the standard designs, as evidenced by their smaller RMSEs. The results underscore that 3PND possesses a desirable property for balancing safety (exploitation) and accuracy (exploration) through the homogeneity constants $\gamma$ or feasibility bound $\alpha$.

### Application to find an optimal $\text {O}^{6}$-BG dose

#### Outline of application

We have illustrated the use of continuous outcomes to indicate the degree of severity of toxicity in the context of oncology trials for finding the MTD for chemotoxic agents. One typical example is a continuous toxicity score measured based on grade information from multiple adverse events [14–16]. However, our modeling framework can be generalized to molecularly targeted agents that have little or no toxicity in the therapeutic dose range. Typical examples include biomarker responses of molecularly targeted agents (see [62] for more details). For example, such a biomarker response might be based on the level of a molecular target, or the change in the level of a target that suggests clinical promise. Basically, this generalization is possible because our modeling framework is based on the principle of the monotonic relationship between a continuous response and a dose.

In the following, we develop a re-design of a dose escalation trial based on a molecularly targeted endpoint, utilizing the dose-response dataset resampled from Friedman et al. [38]. In their study, the authors conducted a phase I trial involving a molecularly targeted agent, $\text {O}^{6}$-benzylguanine ($\text {O}^{6}$-BG). The escalation strategy in this trial was grounded on the reduction of the target enzyme $\text {O}^{6}$-alkylguanine-DNA alkyltransferase (AGT) activity. The patient responses were initially recorded as continuous measurements—specifically, tumor AGT activity measured in fmol/mg—corresponding to discrete dose levels of 40, 60, 80, 100, and 120 $\text {mg}/\text {m}^{2}$ (refer to Table 1 in [38]). The plan involved treating up to 13 patients at each dose level. Despite the continuous nature of the patients’ outcomes, the authors dichotomized these outcomes to facilitate the dose escalation procedure in the trial. Authors employed a rule-based design based on the depletion of the target AGT activity and concluded that a 100 $\text {mg}/\text {m}^{2}$ dose of $\text {O}^{6}$-BG is an optimal dose that will be used in another phase I trial.

**Fig. 5:**
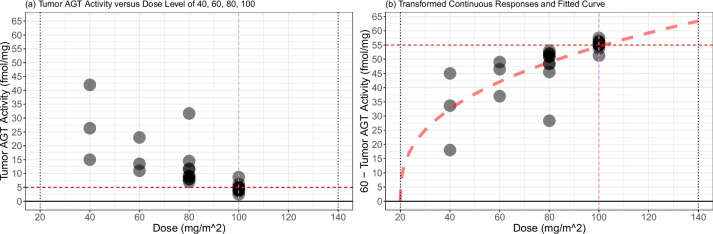
Dose-response relationship of tumor AGT activity versus $\text {O}^{6}$-BG dose levels of 40, 60, 80, an 100 $\text {mg}/\text {m}^{2}$ from 24 patients (**a**) and transformed responses and fitted curve $y = 10.57 \cdot (x - 20)^{0.37}$ (**b**). Horizontal dashed lines on (**a** and **b**) represent 5 fmol/mg and 55 = 60 − 5 fmol/mg, respectively

Figure 5a displays the values of tumor AGT activity from 24 patients: 3, 3, 9, and 9 patients at doses 40, 60, 80, and 100 $\text {mg}/\text {m}^{2}$, respectively. No patients in the trial were assigned to the dose level of 120 $\text {mg}/\text {m}^{2}$. These values are as follows:

- Tumor AGT activity (fmol/mg) of patients assigned to dose 40 $\text {mg}/\text {m}^{2}$: 26.35, 42.00, 15.00.
- Tumor AGT activity (fmol/mg) of patients assigned to dose 60 $\text {mg}/\text {m}^{2}$: 23.00, 13.50, 11.00.
- Tumor AGT activity (fmol/mg) of patients assigned to dose 80 $\text {mg}/\text {m}^{2}$: 31.67, 8.00, 9.00, 14.50, 11.50, 7.00, 11.70, 9.03, 8.00.
- Tumor AGT activity (fmol/mg) of patients assigned to dose 100 $\text {mg}/\text {m}^{2}$: 4.07, 5.00, 8.70, 2.50, 4.07, 6.13, 3.60, 5.00, 5.00.

Among the 24 patients, there are 4 patients whose responses are smaller than 5 fmol/mg, resulting in undetectable tumor AGT activities. These values will later be used in the re-design based on 3PND and EWOC to construct a hypothetical dataset that resembles the actual trial.

In the following, we employ 3PND and EWOC to determine the optimal dose of $\text {O}^{6}$-BG, resulting in undetectable tumor AGT levels (< 5 fmol/mg), as similarly researched by [35].

#### Finding an optimal $\text {O}^{6}$-BG dose using 3PND

In order to apply 3PND for determining an optimal dose of $\text {O}^{6}$-BG, we perform a transformation on the actual values of tumor AGT activity, yielding “60 - Tumor AGT activity,” which will be used as the continuous response in 3PND. This transformation is crucial to establish a monotonic relationship between the continuous response *y* and the dose *x* to implement a model-based design.

In Fig. 5b, we present the transformed dataset alongside the fitted curve (represented by a red dashed line) derived using the least-squares method. Our assumption is that the true dose-response function adheres to the form $y = \beta \cdot (x - 20)^{\nu }$. The estimated parameter values are $\hat{\beta } = 10.57$ and $\hat{\nu } = 0.37$. The non-linear nature of the curve underscores the necessity of incorporating flexibility into the dose-response relationship within our model design.

We apply a dose-escalation rule based on 3PND (Algorithm 2), incorporating two options: “Discrete Dose Selection” and “Monotonic-increasing Dose Sequence.” (Details on the options are described in Discussion.) To simulate a hypothetical dataset for the dose-escalation procedure and leverage the reported data from [38], we introduce perturbation errors sampled from a uniform distribution with specified lower and upper bounds: ±8, ±6, ±4, and ±2 (fmol/mg) corresponding to the dose level of 40, 60, 80, and 100 $\text {mg}/\text {m}^{2}$. (Observing the actual dataset from [38], it is evident that the standard deviation of responses decreases as the dose increases.) These perturbations are correspondingly added to the resampled tumor AGT activity responses. This process constructs a dataset used to assess and refine our dose-finding strategy. We construct CSAD with cohort size of three with accelerated and standard dose-escalation. Variables are specified as follows:

- Number of patients and cohort size: $N = 30$ and $C = 3$.
- Variables specific to accelerated designs: $\alpha = 0.1$.
- Variables specific to standard designs: $\alpha = 0.05$.
- Variables shared in accelerated and standard designs: $\delta = 0.5$, $\gamma = 0.6$, $\eta =55$, $(x_{\text {min}},x_{\text {max}})=(20,140)$, and $x_{1}=40$.

**Table 2:** Results of dose-finding using 3PND

	3PND (accelerated design, $\alpha = 0.1$)			3PND (standard design, $\alpha = 0.05$)		
Patient	Dose ($\text {mg}/\text {m}^2$)	AGT activity (fmol/mg)	Continuous response	Dose ($\text {mg}/\text {m}^2$)	AGT activity (fmol/mg)	Continuous response
1	40	19.82	40.18	40	19.82	40.18
2	40	46.77	13.23	40	46.77	13.23
3	40	8.63	51.37	40	8.63	51.37
4	60	26.98	33.02	40	31.66	28.34
5	60	5.67	54.33	40	7.90	52.10
6	60	8.18	51.82	40	34.90	25.10
7	60	25.07	34.93	60	25.07	34.93
8	60	12.27	47.73	60	12.27	47.73
9	60	9.75	50.25	60	9.75	50.25
10	80	10.81	49.19	60	7.24	52.76
11	80	6.49	53.51	60	25.87	34.13
12	80	9.92	50.08	60	10.81	49.19
13	80	9.72	50.28	60	16.09	43.91
14	80	12.16	47.84	60	27.74	32.26
15	80	30.06	29.94	60	8.59	51.41
16	80	12.46	47.54	60	24.44	35.56
17	80	5.54	54.46	60	9.81	50.19
18	80	4.18	55.82	60	5.26	54.74
19	80	11.05	48.95	80	11.05	48.95
20	80	9.36	50.64	80	9.36	50.64
21	80	15.78	44.22	80	15.78	44.22
22	80	15.93	44.07	80	8.37	51.63
23	80	9.95	50.05	80	16.01	43.99
24	80	9.67	50.33	80	17.81	42.19
25	100	4.30	55.70	80	11.95	48.05
26	100	7.59	52.41	80	9.93	50.07
27	100	9.14	50.86	80	9.87	50.13
28	100	6.10	53.90	80	10.20	49.80
29	100	5.90	54.10	80	9.80	50.20
30	100	3.41	56.59	80	16.32	43.68

Note: The target AGT activity is 5 fmol/mg protein. Continuous responses are obtained by “60 - AGT activity.” Dose assigned to the last cohort of patients is the final estimate of an optimal dose for each design: 100 $\text {mg}/\text {m}^2$ for accelerated design and 80 $\text {mg}/\text {m}^2$ for standard design. Tumor AGT activities (fmol/mg) were resampled from Friedman et al. [38]

**Fig. 6:**
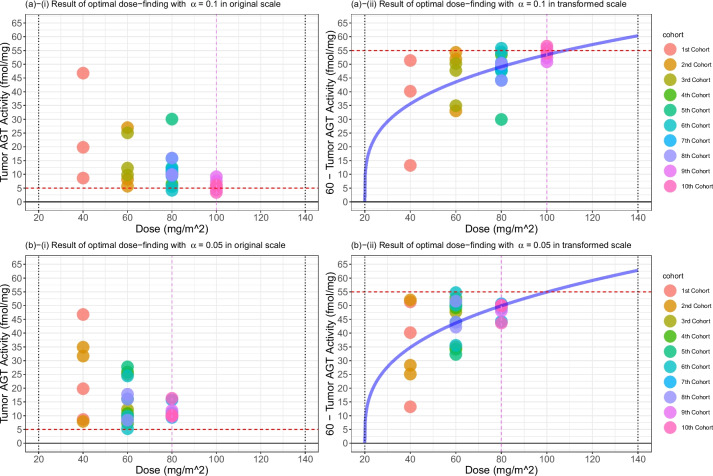
Results of dose-finding using 3PND: accelerated design in (**a**) (i)–(ii) and standard design in (**b**) (i)–(ii). The red vertical dashed lines indicate the final estimates of optimal doses: 100 $\text {mg}/\text {m}^{2}$ for the accelerated design and 80 $\text {mg}/\text {m}^{2}$ for the standard design. The blue curves in panels represent the posterior means of the dose-toxicity curves based on 3PND

Figure 6 displays the results of the dose-escalation based on 3PND designs. Panels a (i)–(ii) and panels b (i)–(ii) correspond to accelerated and standard designs, respectively. Blue curves in panels represent the posterior means of the dose-toxicity curve based on 3PND. Continuous responses and doses are reported in Table 2. The accelerated design ends up finding an optimal dose of 100 $\text {mg}/\text {m}^{2}$, consistent with the findings from [38] and [35]. On the other hand, the standard design selects 80 $\text {mg}/\text {m}^{2}$, which is more conservative than the accelerated design. No patients were overdosed in either design. In the accelerated design, 3 out of 30 patients reported tumor AGT activity smaller than 5 fmol/mg. In contrast, none of the patients in the standard design reported tumor AGT activity smaller than 5 fmol/mg.

The result of a redesign proposed by Wang and Ivanova [35] led to three patients being assigned a dose level of 120 $\text {mg}/\text {m}^{2}$, resulting in overdosing. Since no patients were overdosed using the 3PND, our designs are safer than the similar research conducted previously. Overall, we observe that the 3PND with accelerated dose-escalation shows promise in identifying an optimal $\text {O}^{6}$-BG dose for the current scenario.

#### Comparison with finding an optimal $\text {O}^{6}$-BG dose using EWOC

**Fig. 7:**
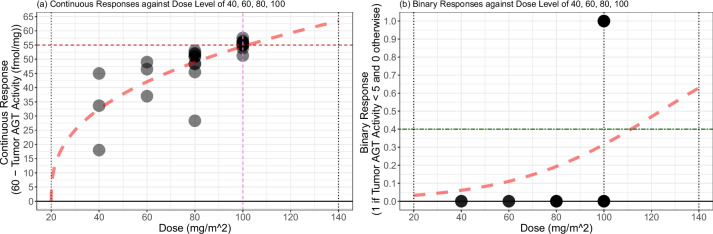
Continuous responses used for 3PND design (**a**) and binary responses used for EWOC design (**b**). Binary responses are obtained by dichotomizing the continuous responses with the threshold value of 55, noted in the red dashed horizontal line in (**a**). Green dashed horizontal line in (**b**) represents the target rate of patients with undetectable tumor AGT activity (that is, $\theta = 0.4 = 1-\gamma$). Red dashed curves on (**a** and **b**) represent fitted dose-toxicity curves based on 3PND and EWOC, respectively

We proceed to apply EWOC [40] in order to utilize the binary responses. For the application of EWOC, we dichotomize the continuous responses (i.e., 60 - tumor AGT activity) using a threshold value of 55 (fmol/mg). As a result of this dichotomization, the binary responses yield $y=1$ when tumor AGT activity is less than 5 fmol/mg, and 0 otherwise. The result of dichotomization process on the continuous response is visually represented in Fig. 7.

**Fig. 8:**
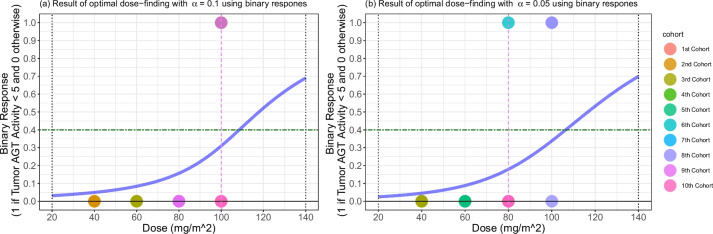
Results of dose-finding using EWOC: accelerated design in (**a**) (i)–(ii) and standard design in (**b**) (i)–(ii). The red vertical dashed lines indicate the final estimates of optimal doses: 100 $\text {mg}/\text {m}^{2}$ for the accelerated design and 80 $\text {mg}/\text {m}^{2}$ for the standard design. The blue curves in panels represent the posterior means of the dose-toxicity curves based on EWOC

To implement EWOC, we use a built-in R function ewoc_d1classical(.) within an R package library(ewoc), with the following set-up: target rate of patients with undetectable tumor AGT activity to be theta = 0.4, the minimum dose to be min_dose = 20, the maximum dose to be max_dose = 140 , the initial dose to be first_dose = 40, and feasibility bound for accelerated design to be alpha = 0.1 and feasibility bound for standard design to be alpha = 0.05, with the specification of uniform prior distributions for the parameters involved in EWOC (See help(ewoc_d1classical) for details about the variable set-up). To simulate a hypothetical dataset with number of patients 30 with cohort size of 3 ($N=30$ and $C=3$), we use the same method adopted in the 3PND re-design, followed by dichotomization with threshold values of 55, as illustrated above. Eventually, this will lead to two EWOC designs with accelerated and standard dose-finding rules, such that the general set-up will be similar to the two 3PND designs with accelerated and standard dose-finding rules, respectively, with the crucial difference being the data form (continuous versus binary responses).

**Table 3:** Results of dose-finding using EWOC

Patient	EWOC (accelerated design, $\alpha = 0.1$)			EWOC (standard design, $\alpha = 0.05$)		
	Dose ($\text {mg}/\text {m}^2$)	AGT activity (fmol/mg)	Binary response	Dose ($\text {mg}/\text {m}^2$)	AGT activity (fmol/mg)	Binary response
1	40	19.81	0	40	19.81	0
2	40	46.77	0	40	46.77	0
3	40	8.62	0	40	8.62	0
4	40	31.66	0	40	31.66	0
5	40	7.89	0	40	7.89	0
6	40	34.90	0	40	34.90	0
7	60	25.06	0	40	29.10	0
8	60	12.27	0	40	40.36	0
9	60	9.74	0	40	13.32	0
10	80	10.81	0	60	7.23	0
11	80	6.49	0	60	25.87	0
12	80	9.91	0	60	10.80	0
13	80	9.72	0	60	16.08	0
14	80	12.16	0	60	27.74	0
15	80	30.06	0	60	8.58	0
16	100	4.54	1	80	12.45	0
17	100	3.77	1	80	5.54	0
18	100	3.08	1	80	4.17	1
19	80	11.05	0	80	11.05	0
20	80	9.36	0	80	9.36	0
21	80	15.77	0	80	15.77	0
22	80	15.92	0	100	3.21	1
23	80	9.94	0	100	2.72	1
24	80	9.67	0	100	5.83	0
25	100	11.95	0	80	11.95	0
26	100	9.92	0	80	9.92	0
27	100	9.87	0	80	9.87	0
28	100	6.09	0	80	10.19	0
29	100	5.90	0	80	9.80	0
30	100	3.41	1	80	16.32	0

Note: The target AGT activity is 5 fmol/mg protein. Binary responses are obtained by 1 if AGT activity < 5, and 0 otherwise

Figure 8 displays the results of dose-finding based on EWOC designs. Panels a (i)–(ii) and panels b (i)–(ii) correspond to accelerated and standard designs, respectively. The blue curve in the panels represents the posterior mean of the dose-toxicity curve based on EWOC design. Binary responses and doses are reported in Table 3. Both the accelerated and standard designs end up identifying optimal doses of 100 $\text {mg/m}^{2}$ and 80 $\text {mg/m}^{2}$, respectively, consistent with the outcomes using 3PND. In the accelerated design, 4 out of 30 patients reported tumor AGT activity smaller than 5 fmol/mg. In the standard design, 3 out of 30 patients exhibited tumor AGT activity smaller than 5 fmol/mg. These patients are indicated by “1” in the binary response column of Table 3.

Overall, we observe that EWOC produces an optimal dose matching that of 3PND. However, the results show that EWOC required a larger number of patients with undetectable tumor AGT activity compared to 3PND. Our results imply that EWOC using binary responses might necessitate a larger patient sample than 3PND using continuous response to effectively learn the dose-response curve.

## Discussion

Modern dose-finding studies and designs are highly specific to individual clinical settings. For example, clinicians may wish to estimate the MTD more precisely by accommodating a particular shape of the dose-toxicity curve or by incorporating information from multiple groups in adaptive designs, etc. Additionally, implementing a dose-finding design for actual phase I cancer clinical trials involves several practical requirements. For instance, it is conventional to have discrete dose levels while allowing only one dose level increment for each patient or cohort. The current section presents several extensions of the basic modeling framework to accommodate such complexities and requirements, thus making the dose selection schemes more realistic.

### Different non-linear dose-toxicity curve

The likelihood part (6) of our design assumes that the relationship between dose and toxicity response can be captured by a power function. The mean function part can be replaced and generalized to a more complex growth curve shape, such as the Richards growth curve [63] or the Gompertz growth curve [64], each having a greater number of parameters than a power function used in 3PND. Such curves can describe a plateau on the dose-toxicity curve so that higher doses may not improve clinical benefit and toxicity does not necessarily increase with increased doses. While these curves would more dynamically describe the dose-toxicity curve than using the power curve, specifying prior distributions requires extensive research to ensure plausibility under small sample problems. Related research based on binary response can be found in [43].

### Accommodation of multiple groups

The model discussed so far assumes that all patients are grouped together, implying that the MTD is expected to be the same for all of them. However, there are circumstances where patients need to be treated in different groups, leading to different MTDs across these groups. Simultaneously, given the limited sample size inherent to dose-finding problems in phase I studies, it is desirable to estimate these MTDs using a single model that allows information to be shared across different groups. The objective within this formulation is to maximize the therapeutic effect of a treatment for individual patients/groups, which is referred to as personalized maximum tolerated doses (MTDs) [65].

Our framework can be extended to estimate MTDs for multiple groups. To illustrate, we consider two groups, labeled as A and B, with the goal of estimating MTDs denoted as $\text {MTD}_{\text {A}}$ and $\text {MTD}_{\text {B}}$. For simplicity, we assume that the curvature parameter $\nu$ is the same and known between the two groups. One approach is to allow the slope parameter to differ while maintaining a shared standard deviation between the groups. Consequently, the likelihood of the extended model will be $y = \beta _{\text {A}}(x - x_{\text {min}})^{\nu } + \sigma \epsilon$ for group A and $y = \beta _{\text {B}}(x - x_{\text {min}})^{\nu } + \sigma \epsilon$ for group B. Conditional priors for $\beta _{\text {A}}$ and $\beta _{\text {B}}$ given $\sigma$, and the prior for $\sigma$, remain the same as Eqs. (7) to (8), respectively. This implies $\beta _{\text {A}}, \beta _{\text {B}}|\sigma \sim \mathcal {U}(l(\sigma ,\nu ),u(\sigma ,\nu ))$ and $\sigma \sim \pi (\sigma ) = \mathcal {C}^{+}(0,1) \mathcal {I}_{(0,\eta /\Phi ^{-1}(\gamma ))}$. Sharing the same standard deviation $\sigma$ between the two groups is crucial; otherwise, the estimation of MTDs becomes parallel, and information borrowing does not occur. With this suggested extension, explicit formulae for the MTDs are $\text {MTD}_{\text {A}} = \xi (\beta _{\text {A}}, \sigma ) = x_{\text {min}} + [(\eta - \sigma \Phi ^{-1}(\gamma ))/\beta _{\text {A}}]^{1/\nu }$ and $\text {MTD}_{\text {B}} = \xi (\beta _{\text {B}}, \sigma ) = x_{\text {min}} + [(\eta - \sigma \Phi ^{-1}(\gamma ))/\beta _{\text {B}}]^{1/\nu }$.

### Discrete dose selection

Let $\Omega = \{ d_{k} \in (x_{\text {min}},x_{\text {max}}) | d_1< \cdots < d_K, \, k=1,\cdots ,K \}$ be the set of ordered dose levels selected for a trial. To adapt the vanilla algorithm (Algorithm 2) to incorporate discrete dose selection, we modify the algorithm’s third step by setting $x_{n+1} = \mathcal {D}_{\alpha } (\mathcal {F}_{n}) = \text {argmin}_{k =1,\cdots ,K } | d_{k} - \Pi _{n}^{-1}(\alpha )| \in \Omega$.

### Stopping rule

A stopping rule can be integrated into Algorithm 1 by adding a break statement. This break statement will terminate the for-loop within the algorithm. The specific condition for the break depends on the context of the toxicity response *Y*(*x*). For instance, if *Y*(*x*) represents a continuous toxicity score, clinicians can implement a break condition whenever the number of patients experiencing a CTCAE Grade $\ge 4$ exceeds a pre-specified threshold. This condition ensures that the trial is halted due to safety concerns.

### Monotonic-increasing dose sequence

This option aims to ensure that the dose sequence $(x_{n})$ is monotonically increasing: $x_{1} \le x_{2} \le \cdots \le x_{N}$. To achieve this, the third step in Algorithm 2 is replaced with $x_{n+1} = \mathcal {D}_{\alpha } (\mathcal {F}_{n}) =\text {max} \{x_n, \Pi _{n}^{-1}(\alpha )\}$. It is important to note that this option disables the de-escalation of the dose. Therefore, the choice of the feasibility bound value $\alpha$ becomes crucial.

### Upper bounding dose increment

To safeguard patients from potential overdose, it is possible to impose an upper bound on the dose increment. For this purpose, in the third step of Algorithm 2, we employ $x_{n+1} = \mathcal {D}_{\alpha } (\mathcal {F}_{n}) = \text {min}\{x_n + M, \Pi _{n}^{-1}(\alpha )\}$, where $M > 0$ is a constant. This design ensures that the dose increment remains constrained within an upper limit of *M*.

### Repeated measurement of toxicity response

In cases where each patient is allowed to be assigned multiple doses, our modeling framework can be easily extended to a non-linear mixed-effect modeling framework, or more generally, Bayesian hierarhical modeling framework [66]. Using 3PND as an example, the likelihood will now take the form of $y_{ij}\sim \mathcal {N}(\beta _{i}(x_{ij} - x_{\text {min}})^{\nu _{i}},\sigma ^{2}),$ $(i=1,\cdots ,N;j = 1,\cdots ,M_{i})$, where $y_{ij}$ represents the continuous toxicity response of the *i*-th patient to the *j*-th dose $x_{ij}$. The parameters $\beta _{i}$ and $\nu _{i}$ denote the slope and non-linearity parameter specific to the *i*-th patient, while the standard deviation $\sigma$ is shared across all *N* patients. A joint prior distribution for these parameters can be similarly given as the prior of 3PND (7) – (9) to ensure that the individualized MTD belong to the dose range, while individual model parameters $(\beta _{i},\nu _{i}), (i=1,\cdots ,N)$ follow a population-level model. Under this formulation, the objective is not only to estimate the individualized MTDs for each patient but also to estimate the population MTD, representing the MTD for all patients.

## Conclusions

In this article, we re-examined the dose-finding problem, delving into its foundational aspects and utilizing continuous outcomes. We presented a comprehensive dose-finding analysis using our modeling framework and introduced the novel 3PND dose-finding algorithm. This algorithm, which estimates the non-linearity parameter through a fully Bayesian approach, benefits from a modern sampling technique that enhances the efficiency of posterior computation (refer to the Appendix “Posterior computation” for details). Simulation experiments yielded results suggesting that our modeling framework demonstrates favorable trial operating characteristics and is well-suited for actual phase I studies, ensuring patient safety and efficient convergence of dose sequences to identify the MTD even with reasonably small sample sizes.

Our modeling framework is not only applicable to determining the MTD for cytotoxic agents in first-in-human studies but also proficient in identifying optimal doses for non-cytotoxic agents and animal studies. This adaptability stems from its foundation in the regression framework.

One specific context where our modeling framework finds relevance is in the domain of molecularly targeted agents. Advances in these agents, characterized by minimal or no toxicity within the therapeutic dose range, have spurred investigations into optimal phase I trial designs [67]. In such scenarios, incorporating pharmacodynamic biomarkers becomes imperative to monitor the drug’s biological effects, particularly in trials involving molecularly targeted agents. Here, the determination of the administered dose is guided by target inhibition considerations rather than toxicity concerns, as elucidated in [62]. As demonstrated in the previous section, the 3PND-based dose-escalation rule can identify optimal doses by accounting for the non-linearity of the dose-response curve.

Another pertinent application lies in pre-clinical studies, particularly those involving repeated doses in individual patients [68]. In such instances, our modeling framework seamlessly extends to a non-linear mixed-effect modeling approach [66]. This extension accommodates multiple doses for each patient, empowering the determination of personalized MTDs for every individual, as outlined in Discussion.

## Figures and Tables

**Figure:**
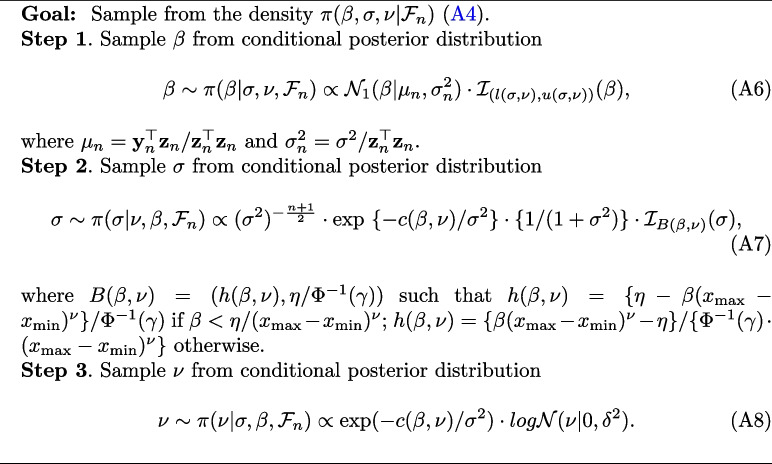
**Algorithm 3** Gibbs sampler

**Figure:**
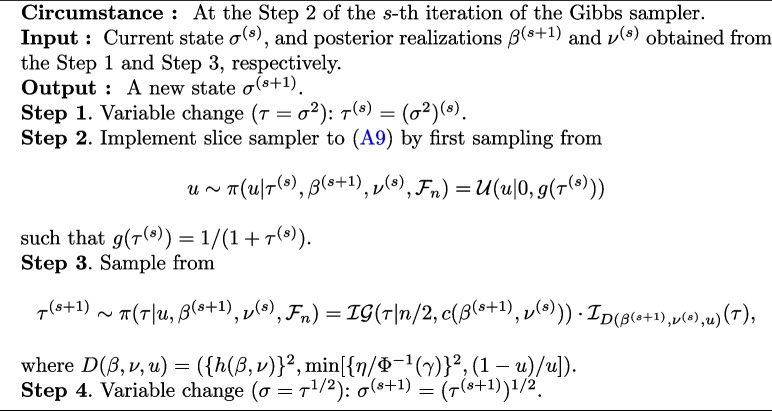
**Algorithm 4** Slice sampler for Step 2

**Figure:**
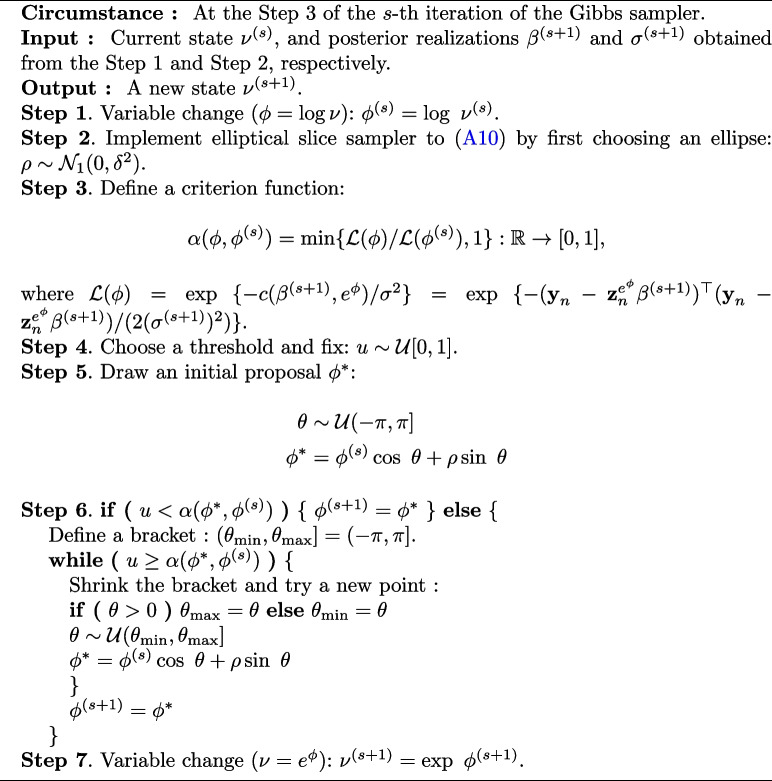
**Algorithm 5** Elliptical slice sampler for Step 3

## Data Availability

The research used simulated data.

## Appendix

### Proof of Theorem 1

We can simplify the inequality (1) as follows

11 $$\begin{aligned} \textbf{Pr}[Y(X) \le \eta | X = x]{} & {} = \textbf{Pr}\left[ \frac{Y(X) - \beta (X - x_{\text {min}})^{\nu }}{\sigma } \le \frac{\eta - \beta (X - x_{\text {min}})^{\nu }}{\sigma } | X = x \right] \nonumber \\{} & {} = \Phi \left( \frac{\eta - \beta (x - x_{\text {min}})^{\nu }}{\sigma } \right) \ge \gamma , \end{aligned}$$

where $\Phi$ denotes the cumulative distribution function of standard normal distribution. By using elementary calculus on the last inequality (11), we have

12 $$\begin{aligned} x \le x_{\text {min}} + \left( \frac{\eta - \sigma \Phi ^{-1}(\gamma )}{\beta } \right) ^{1/\nu }, \end{aligned}$$

The inequality (12) implies that the value $\xi = x_{\text {min}} + [\{\eta - \sigma \Phi ^{-1}(\gamma )\}/\beta ]^{1/\nu }$ is the largest value that satisfies the inequality (11). On the other hand, the following inequality holds due to the parameter constraints on the $(\beta ,\nu ,\sigma )$ (that is, $\beta > (\eta - \sigma \Phi ^{-1}(\gamma )/(x_{\text {max}} - x_{\text {min}})^{\nu }$, $\nu > 0$, and $0< \sigma < \eta /\Phi ^{-1}(\gamma )$), we have $x_{\text {min}}< \xi < x_{\text {max}}$. The two derived inequalities implies that $\xi = x_{\text {min}} + [\{\eta - \sigma \Phi ^{-1}(\gamma )\}/\beta ]^{1/\nu }$ is the largest value that satisfies the inequality (2).

### Proof of Theorem 2

To prove the theorem, we first show that the marginal prior density of MTD $\xi$, denoted as $\pi (\xi )$, is supported within the dose range $(x_{\text {min}}, x_{\text {max}})$, which encompasses the complete parameter space of MTD $\xi$. To this end, we begin by defining $Z = \xi |\sigma ,\nu$ as the continuous random variable representing MTD $\xi$, conditioned on the standard deviation $\sigma$ and the non-linearity parameter $\nu$. We can derive the closed-form expression of the cumulative distribution function of *Z* as follows:

$$\begin{aligned} \nonumber G_{Z}(x){} & {} = \textbf{Pr}[\xi \le x | \sigma ,\nu ] \\{} & {} = \textbf{Pr}\left[ x_{\text {min}} + \left( \frac{\eta - \sigma \Phi ^{-1}(\gamma )}{\beta } \right) ^{1/\nu }\le x |\sigma ,\nu \right] \\{} & {} =\textbf{Pr}\left[ \beta \ge \frac{\eta - \sigma \Phi ^{-1}(\gamma )}{(x - x_{\text {min}})^{\nu }} | \sigma ,\nu \right] \\{} & {} = \frac{u(\sigma ,\nu ) - \{\eta - \sigma \Phi ^{-1}(\gamma )\}/(x - x_{\text {min}})^{\nu } }{u(\sigma ,\nu ) - l(\sigma ,\nu )}\\{} & {} = \frac{(x_{\text {max}} - x_{\text {min}})^{\nu } }{ (x_{\text {max}} - x_{\text {min}})^{\nu } +1} \cdot \frac{1}{\sigma \Phi ^{-1}(\gamma )} \cdot \left( \frac{\eta }{(x_{\text {max}} - x_{\text {min}})^{\nu }} + \sigma \Phi ^{-1}(\gamma ) - \frac{\eta - \sigma \Phi ^{-1}(\gamma )}{(x - x_{\text {min}})^{\nu }}\right) . \end{aligned}$$

It is straightforward to show that $G_{Z}(x)$ has the following properties: (i) $G_{Z}(x)$ is strictly increasing on the open interval $(x_{\text {min}}^{*}(\sigma ,\nu ) ,x_{\text {max}})$, (ii) $G_{Z}(x_{\text {min}}^{*}(\sigma ,\nu )) = 0$, and (iii) $G_{Z}(x_{\text {max}}) = 1$, where the left end point $x_{\text {min}}^{*}(\sigma ,\nu )$ of the interval is given by

13 $$\begin{aligned} x_{\text {min}}^{*}(\sigma ,\nu ) = x_{\text {min}} + \left[ \frac{\{\eta - \sigma \Phi ^{-1}(\gamma )\}\cdot (x_{\text {max}} - x_{\text {min}})^{\nu }}{\eta + \sigma \Phi ^{-1}(\gamma )\cdot (x_{\text {max}} - x_{\text {min}})^{\nu }}\right] ^{1/\nu }. \end{aligned}$$

Therefore, the random variable $Z = \xi |\sigma ,\nu$ is supported on the interval $(x_{\text {min}}^{*}(\sigma ,\nu ) ,x_{\text {max}})$.

Note that the left end point $x_{\text {min}}^{*}(\sigma ,\nu )$ is a function of $\sigma$ and $\nu$, whose support is given as $(0, \eta /\Phi ^{-1}(\gamma )) \times (0,\infty )$. On the other hand, by using elementary calculus, we can show that the function $x_{\text {min}}^{*}(\sigma ,\nu )$ (13) is monotonically deceasing and continuous on the interval $(0, \eta /\Phi ^{-1}(\gamma ))$, and the following one-sided limits hold: $\text {lim}_{\sigma \rightarrow (\eta /\Phi ^{-1}(\gamma ))^{-}}x_{\text {min}}^{*}(\sigma ,\nu ) = x_{\text {min}} \text { and } \text {lim}_{\sigma \rightarrow 0^{+}}x_{\text {min}}^{*}(\sigma ,\nu ) = x_{\text {max}}$, for any fixed value of $\nu >0$. Summing the results indicates that the marginal random variable $\xi$ is supported on the open interval $(x_{\text {min}},x_{\text {max}})$.

Because the marginal prior of $\xi$ is compactly supported on the open interval $(x_{\text {min}},x_{\text {max}})$, the measure zero set over the dose range $(x_{\text {min}},x_{\text {max}})$ with respect to the prior measure $\pi (\xi )$ is the empty set. Thus, by the Doob’s theorem [56], the posterior distribution of $\xi$ is consistent at any value $\xi _0 \in (x_{\text {min}},x_{\text {max}})$.

### Posterior computation

#### Gibbs sampler

Let $\mathcal {F}_n = \{(x_i, y_i)\}_{i=1}^{n}$ denote the set of the *n* observations from the first *n* subjects, where $y_i = Y(x_i)$ represents the continuous toxicity response of the *i*-th patient against the assigned dose $x_i$. We consider a vector-form of the 3PND (6) – (9): ${\textbf {y}}_n = {\textbf {z}}_n ^{\nu } \beta + \sigma \varvec{\epsilon }_{n}$, $\varvec{\epsilon }_{n}\sim \mathcal {N}_{n}({\textbf {0}} ,{\textbf {I}}_{n})$ (6), where ${\textbf {y}}_{n} = (y_1, \cdots , y_n)^{\top }$ and ${\textbf {z}}_{n}^{\nu } = ((x_1 - x_{\text {min}})^{\nu }, \cdots , (x_n - x_{\text {min}})^{\nu })^{\top }$ are *n*-dimensional response and covariate vectors, respectively. Here, the $\nu$ in the vector notation ${\textbf {z}}_{n}^{\nu }$ implies the component-wise exponent.

Now, our eventual goal is to simulate a sample from the joint posterior distribution

14 $$\begin{aligned} \pi (\beta , \nu , \sigma | \mathcal {F}_n ) = \frac{\mathcal {N}_{n}({\textbf {y}}_n | \textbf{z}_n^{\nu } \beta , \sigma ^{2} \textbf{I}_{n})\cdot \pi (\beta ,\nu ,\sigma ) }{ \int _{l(\sigma ,\nu )}^{u(\sigma ,\nu )} \int _{0}^{\infty } \int _{0}^{\eta / \Phi ^{-1}(\gamma )} \mathcal {N}_{n}({\textbf {y}}_n | \textbf{z}_n^{\nu }\beta , \sigma ^{2} \textbf{I}_{n})\cdot \pi (\beta ,\nu ,\sigma ) d\sigma d\nu d \beta }, \end{aligned}$$

where $$l(\sigma ,\nu ) = \{\eta - \sigma \Phi ^{-1}(\gamma )\}/(x_{\text {max}} - x_{\text {min}})^{\nu }$$ and $$u(\sigma ,\nu ) =\{\eta /(x_{\text {max}} - x_{\text {min}})^{\nu }\} + \sigma \Phi ^{-1}(\gamma )$$.

The nominator of the joint density $\pi (\beta , \nu , \sigma | \mathcal {F}_n )$ (14) is detailed as

15 $$\begin{aligned} \nonumber{} & {} \mathcal {N}_{n}\left({\textbf {y}}_n | \textbf{z}_n^{\nu } \beta , \sigma ^{2} \textbf{I}_{n}\right) \cdot \pi (\beta |\sigma ,\nu ) \cdot \pi (\sigma ) \cdot \pi (\nu )\\ \nonumber{} & {} = \mathcal {N}_{n}({\textbf {y}}_n | \textbf{z}_n \beta , \sigma ^{2} \textbf{I}_{n}) \cdot \mathcal {U}(\beta |l(\sigma ,\nu ),u(\sigma ,\nu )) \cdot \mathcal {C}^{+}(\sigma |0,1) \mathcal {I}_{(0,\eta /\Phi ^{-1}(\gamma ))}(\sigma ) \cdot log \mathcal {N}(\nu |0,\delta ^{2}) \\{} & {} \propto \frac{1}{(\sigma ^{2})^{n/2}} \exp \ \left( - \frac{c(\beta ,\nu )}{\sigma ^2} \right) \cdot \frac{1}{\sigma } \mathcal {I}_{(l(\sigma ,\nu ),u(\sigma ,\nu ) )}(\beta ) \cdot \frac{1}{1+\sigma ^2} \mathcal {I}_{(0,\eta /\Phi ^{-1}(\gamma ))}(\sigma ) \cdot \frac{1}{\nu } \exp \left( - \frac{(\log \nu )^{2}}{2 \cdot \delta ^{2}}\right) , \end{aligned}$$

where $c(\beta ,\nu ) = ({\textbf {y}}_{n} - {\textbf {z}}_{n}^{\nu } \beta )^{\top } ({\textbf {y}}_{n} - {\textbf {z}}_{n}^{\nu } \beta )/2$. Because the joint density $\pi (\beta ,\nu ,\sigma |\mathcal {F}_n)$ cannot be obtained in a closed-form distribution, we develop a MCMC algorithm to sample from the joint density. Algorithm 3 details a Gibbs sampler [58, 69] to sample from the density $\pi (\beta ,\nu , \sigma |\mathcal {F}_n)$ (15).

Note that the density $\pi (\beta |\sigma ,\nu , \mathcal {F}_{n})$ (16) in **Step 1** is a truncated normal distribution supported on the interval $(l(\sigma ,\nu ),u(\sigma ,\nu ))$ with mean $\mu _{n}(\nu )$ and variance $\sigma _{n}^{2}(\nu )$. Using a statistical software R, one may use R package truncnorm to sample from it. To implement **Step 2** and **Step 3**, one consideration is to use Metropolis-Hasting algorithm (MH) [57] due to the non-closed form the full conditional densities, which may have an issue of sampling efficiency. Instead, in the next subsections we introduce modern MCMC sampling techniques to sample from the densities.

#### Slice sampler implementation in Step 2

We use the slice sampler [59] to implement **Step 2**. Slice sampler is popular for its ability to adapt the change of the step size based on the local property of the target density. A central idea of the slice sampler is to find a valid parameter expansion from the target density, and use the Gibbs sampler to the expanded target distribution [70]. See [59] for more detail.

To apply the slice sampler in the **Step 2** (17), first, use the change of variable, $\tau = \sigma ^{2}$, to get

19 $$\begin{aligned} \pi (\tau | \beta ,\nu , \mathcal {F}_n ){} & {} =\pi (\sigma | \beta ,\nu , \mathcal {F}_n )|_{\sigma = \tau ^{1/2}} \cdot |d\sigma /d \tau | \nonumber \\{} & {} \propto \tau ^{-(n+1)/2}\exp \ \{- c(\beta ,\nu )/\tau \} \cdot \{1/(1 + \tau ) \} \cdot \mathcal {I}_{B(\beta ,\nu )}(\tau ^{1/2}) \cdot |\tau ^{-1/2}| \nonumber \\{} & {} \propto \tau ^{-n/2-1}\exp \ \{- c(\beta ,\nu )/\tau \} \cdot \{1/(1 + \tau ) \} \cdot \mathcal {I}_{A(\beta ,\nu )}(\tau ) \nonumber \\{} & {} \propto \mathcal{I}\mathcal{G} (\tau | n/2, c(\beta ,\nu )) \cdot g(\tau ) \cdot \mathcal {I}_{A(\beta ,\nu )}(\tau ) \end{aligned}$$

where $g(\tau ) = 1/(1 + \tau )$ and $A(\beta ,\nu ) = ( \{h(\beta ,\nu )\}^2, \{\eta / \Phi ^{-1}(\gamma )\}^2 )$. The notation $\mathcal{I}\mathcal{G}(a,b)$ denotes the inverse gamma distribution with shape parameter *a* and scale parameter *b*. Note that the function $u = g(\tau )$ is decreasing on the positive real line, and its inverse function is given by $\tau = g^{-1}(u) = (1-u)/u$. Consider a joint density, $\pi (\tau , u| \beta ,\nu , \mathcal {F}_n ) \propto \mathcal{I}\mathcal{G} (\tau | n/2, c(\beta ,\nu ) ) \cdot \mathcal {I}_{ (0, g(\tau )) }(u) \cdot \mathcal {I}_{A(\beta ,\nu )}(\tau ).$. We can show that $\int \pi (\tau , u| \beta ,\nu , \mathcal {F}_n ) du = \pi (\tau | \beta ,\nu , \mathcal {F}_n)$, which implies that $\pi (\tau , u| \beta ,\nu , \mathcal {F}_n )$ is a valid parameter expansion of the density $\pi (\tau | \beta ,\nu , \mathcal {F}_n )$ (19). Finally, execute the Gibbs sampler to the joint density $\pi (\tau , u| \beta ,\nu , \mathcal {F}_n )$, and disregard *u*, and transform $\tau$ back to $\sigma$ to get a realization from $\pi (\sigma | \beta ,\nu , \mathcal {F}_n )$.

Algorithm 4 details the steps to implement the slice sampler:

#### Elliptical slice sampler implementation in Step 3

The current subsection illustrates how to sample from the conditional posterior density $\pi ( \nu |\beta ,\sigma , \mathcal {F}_{n})$ (18) by using the elliptical slice sampler (ESS) [60]. Conceptually, ESS and MH algorithms are similar in that both comprises two steps: proposal step and criterion step. A difference between the two algorithms arises in the criterion step. If a new candidate does not pass the criterion, then MH takes the current state as the next state: whereas ESS re-proposes a new candidate until rejection does not take place, rendering the algorithm rejection-free. To apply the ESS in the **Step 3** [18], we start with the change of variable, $\phi = \log \nu$, to get

20 $$\begin{aligned} \nonumber \pi ( \phi |\beta ,\sigma , \mathcal {F}_{n}){} & {} = \pi ( \nu |\beta ,\sigma , \mathcal {F}_{n})|_{\nu = e^{\phi }} \cdot |d\nu /d\phi |\\ \nonumber{} & {} \propto \exp \ \left\{-c(\beta ,e^{\phi })/\sigma ^2 \right\} \cdot \log \mathcal {N}_{1}\left(e^{\phi }|0,\delta ^{2}\right) \cdot e^{\phi }\\{} & {} \propto \mathcal {L}(\phi ) \cdot \mathcal {N}_{1}(\phi |0,\delta ^{2}), \end{aligned}$$

where $\mathcal {L}(\phi ) = \exp \ \{-c(\beta ,e^{\phi })/\sigma ^2 \}$. Algorithm 5 details the steps to implement ESS:

## Abbreviations

MTD: Maximum tolerated dose
DLT: Dose-limiting toxicity
CTCAE: Common toxicity criteria for adverse events
NPD: Number of patients with dose-limiting toxicity
NPO: Number of patients overdosed
BTM: Bias to MTD
RMSE: Square root of the relative mean squared error
3PND: Three-parameter non-linear dose-finder
EWOC: Escalation with overdose control

## Code availability

Details of posterior computation are described in the Appendix.
